# The Synthesis and Properties of Ladder-Type π-Conjugated Compounds with Pyrrole and Phosphole Rings

**DOI:** 10.3390/molecules29010038

**Published:** 2023-12-20

**Authors:** Minh Anh Truong, Suzuho Morishita, Keiichi Noguchi, Koji Nakano

**Affiliations:** 1 Department of Applied Chemistry, Graduate School of Engineering, Tokyo University of Agriculture and Technology, 2-24-16 Naka-cho, Koganei, Tokyo 184-8588, Japan; truong.minhanh.2x@kyoto-u.ac.jp (M.A.T.); suzuho.akiyama@agilent.com (S.M.); 2 Instrumentation Analysis Center, Tokyo University of Agriculture and Technology, 2-24-16 Naka-cho, Koganei, Tokyo 184-8588, Japan; knoguchi@cc.tuat.ac.jp

**Keywords:** phosphole, pyrrole, π-conjugated compound, photoluminescence

## Abstract

The phosphole ring is known as a useful building block for constructing π-conjugated organic materials. Here, we report ladder-type benzophospholo[3,2-*b*]indole (BPI) derivatives where the phosphole and the pyrrole rings are directly fused. Compounds **8a**–**8d** with different aryl groups on the phosphorous center were successfully synthesized, and the solid-state structure of **8a** was confirmed using X-ray crystallographic analysis. The BPIs exhibit relatively high fluorescence quantum yield (Φ 0.50−0.72) and demonstrate a larger Stokes shift compared with a series of benzophospholo[3,2-*b*]benzoheteroles. The benzophospholo[3,2-*b*]carbazole derivative **9**, which possesses a benzene ring between the phosphole and the pyrrole rings of the BPI, was also synthesized, and its solid-state structure was confirmed using X-ray crystallographic analysis. Compound **9** was found to show a smaller Stokes shift compared with the BPI.

## 1. Introduction

Phosphole is recognized as a promising building block for functional π-conjugated organic materials owing to its unique properties [1,2,3,4,5,6,7,8,9]. The orbital interaction of the phosphorous lone pair with the butadiene π-system is inhibited since the phosphorous center offers a pyramidal geometry [10]. On the other hand, there is an effective orbital interaction between the π* orbital of the butadiene moiety and the σ* orbital of the exocyclic P–C bond. Accordingly, phosphole acts as a nonaromatic cyclic diene with a low-lying lowest unoccupied molecular orbital (LUMO). Further electronic and steric tuning can also be achieved through functionalization of the phosphorous center. In particular, the oxidation of the phosphorous center to the phosphine oxide is attractive since the resulting phosphole *P*-oxide can work as an electron-accepting unit in a π-conjugated framework. Based on these attractive features of phosphole, a range of phosphole-containing π-conjugated compounds were developed and their application as organic electronic materials and chromophores was realized.

**Figure 1:**
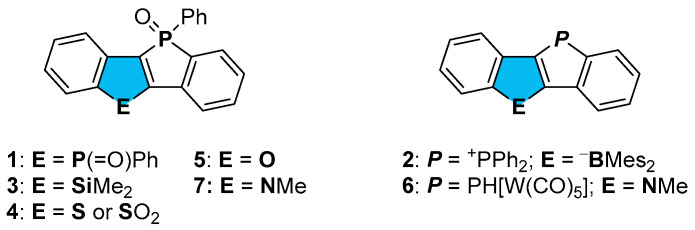
Structures of benzophospholo[3,2-*b*]benzoheterole derivatives.

The introduction of a second heterocycle, such as thiophene and furan, into phosphole-containing π-conjugated skeletons was found to have a great impact on their electronic and optical properties. In the last two decades, a series of ladder-type benzophospholo[3,2-*b*]benzoheteroles, where the phosphole and the heterole rings are directly fused, have been reported as highly emissive materials (Figure 1). For example, Yamaguchi and co-workers developed the benzophospholo[3,2-*b*]benzophosphole derivative **1**, which had an extraordinarily high fluorescence quantum yield (Φ) of 0.99 [11]. Following this report, the borole (**2**) [12,13], silole (**3**) [14,15], and thiophene (**4**) [16,17,18] analogs were reported, exhibiting high fluorescence quantum yield. We also reported the furan analogs, benzophospholo[3,2-*b*]benzofurans (BPBFs), and found that they were highly emissive materials (Φ 0.84−0.90) [19]. It is noteworthy that the furan analog **5** demonstrates the largest Stokes shift (5960 cm^−1^ in CH_2_Cl_2_) among the corresponding benzophospholo[3,2-*b*]benzoheteroles **1** (4480 cm^−1^ in CH_2_Cl_2_), **3** (4550 cm^−1^ in CH_2_Cl_2_), and **4** (4080 cm^−1^ in CH_2_Cl_2_ for absorption spectrum and in cyclohexane for emission spectrum). In addition, the furan analog **5** showed red-shifted photoluminescence spectra with an increase in solvent polarity. The combination of an electron-rich furan unit with an electron-accepting phosphole *P*-oxide unit may result in a significantly large Stokes shift and positive solvatochromism**.** Therefore, we envisaged that the introduction of an electron-rich pyrrole ring should also induce a large Stokes shift. To date, the pyrrole analogs (benzophospholo[3,2-*b*]indoles, BPIs) have been reported from the three research groups. The first example is the tungsten complex **6** in which the lone pair of the phosphole unit coordinates to the tungsten center [20]. However, it is difficult to evaluate the effect of a pyrrole ring based on the photophysical properties of **6**. The tungsten complex **6** possesses a hydride-substituted and tungsten-coordinated phosphole unit, while the reported benzophospholo[3,2-*b*]benzoheteroles possess a phenyl-substituted phosphole *P*-oxide unit. In 2017, the pyrrole analog **7** with a phenyl-substituted phosphole *P*-oxide unit was developed by Muranaka, Yasuike, and co-workers [21]. A large Stokes shift (5950 cm^−1^ in CH_2_Cl_2_) comparable to that of the oxygen analog **5** was demonstrated, while the solvent effect has not been investigated.

**Figure 2:**
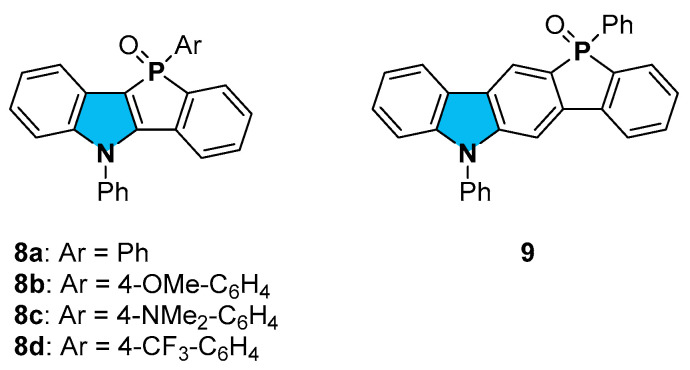
Structures of benzophospholo[3,2-*b*]indole derivatives (BPIs) and their higher homolog.

In this context, we herein report a follow-up study on the synthesis and photophysical properties of BPI **8** (Figure 2). The substituents on the nitrogen and phosphorous centers of **8** are different from those of **7**, thereby allowing us to investigate the structure–property relationship. We also synthesized the higher homolog **9** with a fused benzene ring between the pyrrole and the phosphole units in order to reveal the effect of the direct fusion of the pyrrole and phosphole rings.

## 2. Results

### 2.1. Synthesis

**Scheme 1:**
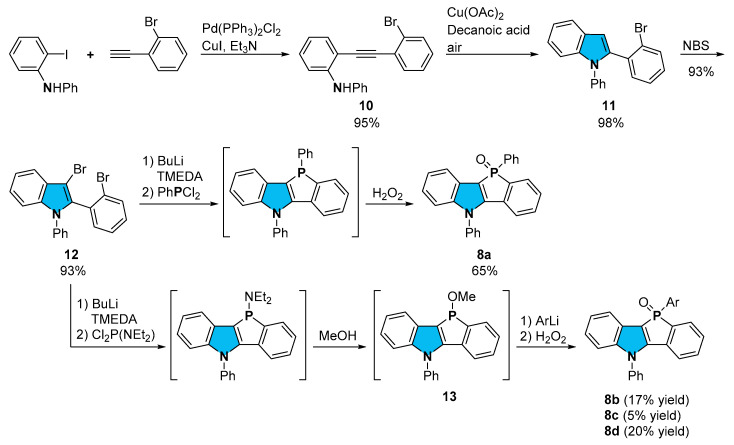
Synthesis of BPI derivatives **8a**–**8d**.

**Figure 3:**
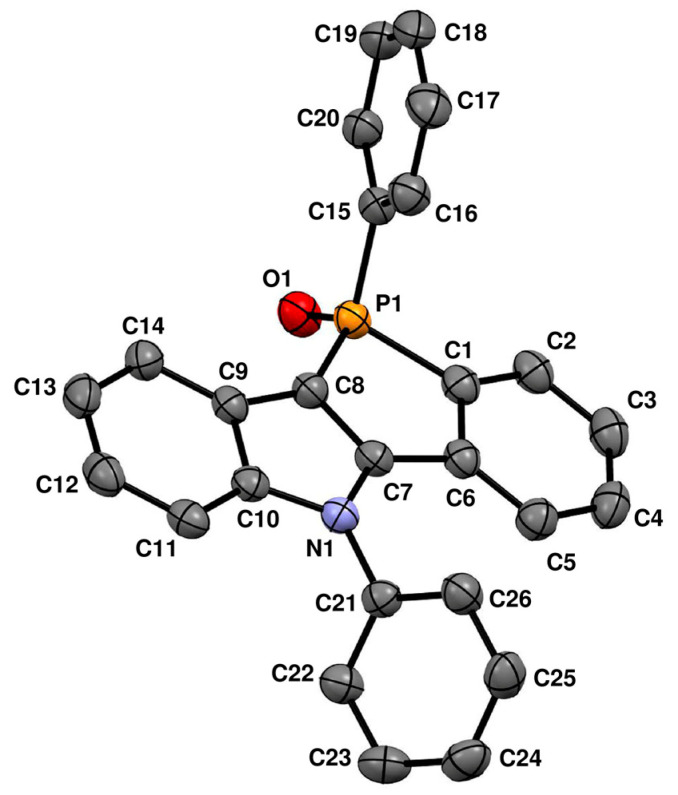
ORTEP drawing of **8a** (50% thermal ellipsoids). All hydrogen atoms are omitted for clarity.

The reaction of 2,2′-dilithiated biaryls with dichlorophosphines is one of the most convenient synthetic routes to (hetero)arene-annulated phosphole [2,7]. Indeed, BPBF **5** in our previous report and the BPI derivative **7** were synthesized by employing dilithiated 2-phenylbenzofuran and 2-phenylindole, respectively [19,21]. Therefore, we designed compound **12** as a starting material for the synthesis of BPI **8** (Scheme 1). Compound **12** was successfully synthesized via three steps. Firstly, the Sonogashira−Hagihara coupling reaction of *N*-(2-iodophenyl)aniline with 1-bromo-2-ethynylbenzene produced diarylethyne **10**. Then, the copper-catalyzed cyclization [22] and the subsequent bromination with *N*-bromosuccinimide (NBS) afforded compound **12**. The obtained compound **12** was treated with BuLi in the presence of *N*,*N*,*N*′,*N*′-tetramethylethylenediamine (TMEDA), and the resulting dilithiated species was trapped using dichlorophenylphosphine. Since the trivalent phosphorous center is easily oxidized under air, the product was isolated as the air-stable phosphole *P*-oxide derivative **8a** after treatment with aqueous H_2_O_2_ (65% yield from **12**). The structure of **8a** was confirmed using X-ray crystallographic analysis (Figure 3). The central π-conjugated core was almost planar (r.m.s. deviation 0.046 Å). The π–π stacking interaction of the central π-conjugated cores was inhibited (interplane distance 4.60 Å), which is due to the steric requirement of the two phenyl groups on the nitrogen and phosphorus atoms. Compound **8a** is a chiral molecule owing to the stereogenic phosphorus atom, and both enantiomers were contained in a unit cell.

The BPI derivatives **8b**–**8d,** with different aryl substituents on the phosphorous center, were also prepared. Previously, we reported a facile synthetic strategy for BPBF derivatives with a variety of aryl groups on the phosphorous center [19]. The same strategy was applied to the synthesis of **8b**–**8d**. The common synthetic intermediate **13** was first prepared via dilithiation of compound **12**, treatment with Cl_2_PNEt_2_, and the subsequent reaction with methanol. Then, the BPI derivatives **8b**–**8d** were obtained using the reaction of compound **13** with the corresponding aryllithium reagent and the following oxidation with aqueous H_2_O_2_.

### 2.2. Photophysical Properties

**Figure 4:**
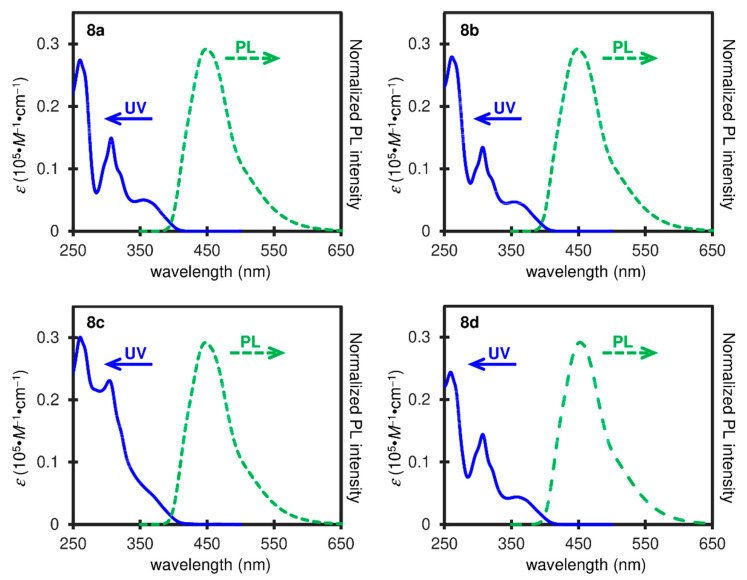
UV–vis absorption and PL spectra of **8a**–**8d** in CH_2_Cl_2_.

The photophysical properties of BPI **8** were evaluated by ultraviolet-visible (UV)−vis absorption and photoluminescence (PL) spectroscopies. Their spectra are shown in Figure 4, and their photophysical data are summarized in Table 1. Compound **8a** showed the longest absorption band with a maximum (λ_abs_) at 355 nm in CHCl_3_ and 353 nm in CH_2_Cl_2_. The spectrum shape is the same as that of its methyl analog **7**, while the longest absorption maximum is slightly blue-shifted compared with that of **7** (355 nm in CH_2_Cl_2_) [21]. For the PL measurements, the BPI derivative **8a** demonstrated the emission maxima (λ_PL_) at 449 nm both in CHCl_3_ and CH_2_Cl_2_. The Stokes shifts of **8a** were estimated to be 5900 cm^−1^ in CHCl_3_ and 6060 cm^−1^ in CH_2_Cl_2_. The Stokes shift in CH_2_Cl_2_ is larger than that of the methyl analog **7** (5950 cm^−1^). Compounds **8a**, **8b**, and **8d** showed almost identical absorption spectra with the longest absorption maxima at 355–358 nm in CHCl_3_. On the other hand, compound **8c** with a 4-(dimethylamino)phenyl group on the phosphorus center showed less resolved absorption spectrum without a distinct maximum at around 350 nm. The PL spectra of BPIs **8a**–**8d** are almost identical with the emission maxima at around 450 nm. The absolute quantum yields (Φ) of **8a**, **8b**, and **8d** are relatively high ≈ 0.70, while the Φ value of **8c** is much smaller (0.50).

The photophysical properties of the BPIs were compared with those of the BPBFs [19]. The longest absorption maxima and emission maxima (in CHCl_3_) of BPIs **8a**, **8b**, and **8d** were red-shifted by 12–13 nm (970–1070 cm^−1^) and 20–21 nm (1020–1090 cm^−1^), respectively, in comparison with the corresponding BPBFs. Compounds **8a** (5900 cm^−1^), **8b** (5900 cm^−1^), and **8d** (5860 cm^−1^) demonstrated slightly larger Stokes shifts than the corresponding BPBFs with phenyl (5790 cm^−1^), anisyl (5880 cm^−1^), and 4-(trifluoromethyl)phenyl (5810 cm^−1^) substituents on the phosphorus center, respectively, which are one of the largest Stokes shifts among the benzophospholo[3,2-*b*]benzoheterole derivatives. Both the BPI and the BPBF derivatives with a 4-(dimethyamino)phenyl group on the phosphorus center demonstrated less resolved absorption spectra. The PL emission maximum of the BPI derivative **8c** is almost identical to those of BPIs **8a**, **8b**, and **8d**, while the BPBF derivative with a 4-(dimethylamino)phenyl group was reported to exhibit a largely red-shifted PL spectrum among a series of BPBFs [19]. Thus, the effect of the 4-(dimethylamino)phenyl group on emission properties is different between BPI and BPBF.

**Figure 5:**
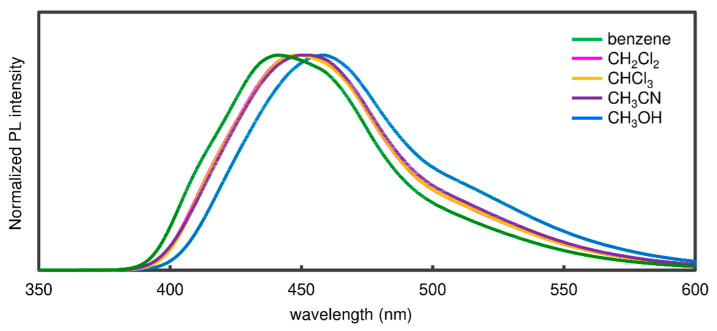
PL spectra of **8a** in various solvents.

The absorption and emission properties of **8a** were also evaluated in different solvents (Table 2). The absorption spectra did not shift significantly in various organic solvents (Figure S22). On the other hand, a solvatochromic shift was observed for the PL spectrum (Figure 5 and Table 1). The emission maximum showed a substantial red-shift with an increase in solvent polarity [23]. The solvatochromic shift of 890 cm^−1^, which was demonstrated by changing the solvents from the less polar benzene to the highly polar methanol, was larger than that of the BPBF **5** (810 cm^−1^) [19].

### 2.3. Theoretical Study on Photophysical Properties

**Figure 6:**
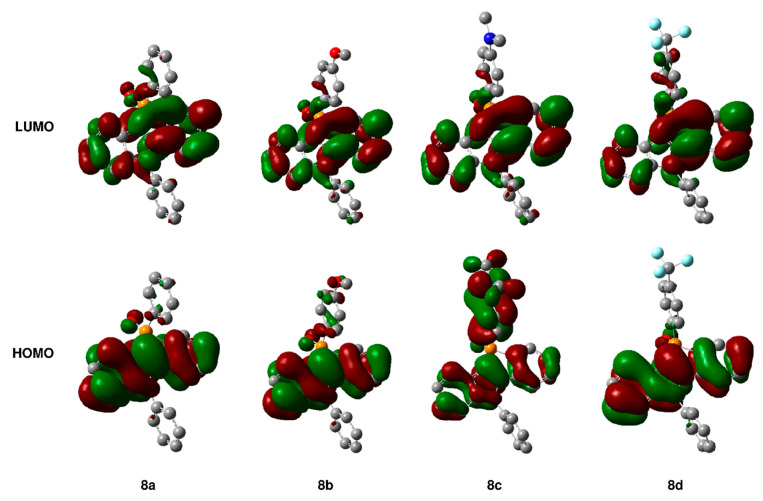
Frontier molecular orbitals of **8a**–**8d**.

The theoretical calculations using density functional theory (DFT) and time-dependent (TD) DFT methods at the B3LYP/6-31+G(d) level of theory were carried out to understand the experimental photophysical properties [24]. The HOMO/LUMO are shown in Figure 6, and the detailed calculation results are given in the Supporting Information. The calculated longest wavelength absorption of **8a** (360 nm) is almost identical to the experimental value (355 nm, Table 1). This S_0_→S_1_ electron transition is dominated by the HOMO−LUMO (π−π*) transition. The HOMO orbital spreads over the π-conjugated core and no contribution of the phosphorus center was demonstrated. On the other hand, the LUMO orbital is largely dominated by the phosphole *P*-oxide moiety. The calculated longest wavelength absorptions of **8b** and **8d** agree well with the experimental absorption maxima. Their S_0_→S_1_ electron transitions are derived from the HOMO−LUMO (π−π*) transition. The HOMO and LUMO distributions of them are similar to those of **8a**. The methoxy and trifluoromethyl groups have little impact on the HOMO and LUMO distributions, and the experimental absorption spectra of BPIs **8a**, **8b**, and **8d** are almost identical as described above. The BPI **8c** gives the calculated longest wavelength absorption at 373 nm, which is red-shifted compared to those of BPIs **8a**, **8b**, and **8d**. However, the oscillator strength is smaller than those of **8a**, **8b**, and **8d**, resulting in a less resolved absorption spectrum. The LUMO distribution of **8c** is similar to those of **8a**, **8b**, and **8d**, while the HOMO distribution is different between them. The HOMOs **8a**, **8b**, and **8d** are mainly located on the π-conjugated core with almost no contribution from the aryl substituents on the phosphorus center. In contrast, the HOMO of **8c** is located both on the π-conjugated core and the 4-(dimethylamino)phenyl substituent, which may cause the less resolved absorption spectrum of **8c** described above. The HOMO distribution of **8c** is also different from that of the oxygen analog BPBF **5** that we previously reported [19]. The HOMO of **5** is dominated by 4-(dimethylamino)phenyl substituent (donor), and the LUMO is localized on the π-conjugated core with a larger contribution from the phosphole *P*-oxide moiety (acceptor). Such HOMO/LUMO distributions would induce an intramolecular charge transfer nature, resulting in a largely red-shifted PL spectrum of **5** among a series of BPBFs. On the other hand, the HOMO of **8c** is distributed both on the π-conjugated core and the 4-(dimethylamino)phenyl substituent. Therefore, the BPI derivative **8c** does not exhibit an intramolecular charge transfer nature and shows almost the same PL spectrum as those of **8a**, **8b**, and **8d**.

### 2.4. Comparison with Higher Homolog

The dibenzo[3,2-*b*]benzodiphosphole derivative, a higher homolog of benzophosphoro[3,2-*b*]benzophosphole, was developed [25,26,27]. The higher homolog of BPI, the benzophospholo[3,2-*b*]carbazole (BPC) derivative, was also synthesized previously via the Suzuki–Miyaura cross-coupling reaction of bromodibenzophosphole with amino-substituted phenylboronic acid, followed by catalytic C–H amination [28]. The substituent on the nitrogen center is a benzyl group, and its photophysical properties were not discussed. Therefore, in order to investigate the effect of direct fusion of pyrrole and phosphole units on photophysical properties, we designed benzophospholo[3,2-*b*]carbazole **9** with a phenyl group on each nitrogen and phosphorus center.

**Scheme 2:**
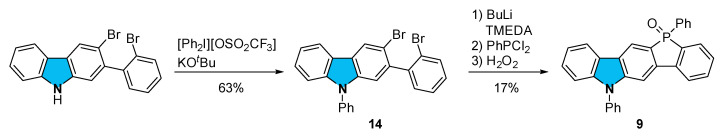
Synthesis of BPC **9**.

**Figure 7:**
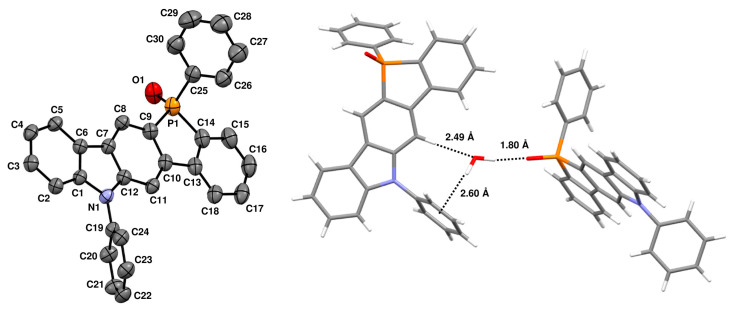
ORTEP drawing of **9** (50% thermal ellipsoids. All hydrogen atoms and water molecules are omitted for clarity.) and the hydrogen bonds between the neighboring two molecules.

The synthesis of **9** is described in Scheme 2. The reaction of 2,2′-dilithiated biaryls with dichlorophenylphosphine was again applied to construct the dibenzophosphole moiety. The starting material **14** for the dibenzophosphole construction was synthesized using transition-metal-free *N*-arylation [29] of 3-bromo-2-(2-bromophenyl)carbazole [30] with diphenyliodonium salt. Treatment of **14** with BuLi, the reaction with PhPCl_2_, and the following oxidation produced the desired compound **9** in 17% yield. The structure of **9** was confirmed using X-ray crystallographic analysis (Figure 7). The π-conjugated core was found to be planar (r.m.s. deviation 0.022 Å), and both enantiomers were contained in a unit cell. The interplane distance between the neighboring molecules is 3.46 Å, indicating the presence of π–π stacking. Water molecules were incorporated in the single crystal and interacted with two BPC molecules via O–H···π(phenyl) (2.60(4) Å) and O–H···O(phosphoryl) (1.80(4) Å) and C–H···O(water) (2.49 Å) hydrogen bonds.

**Figure 8:**
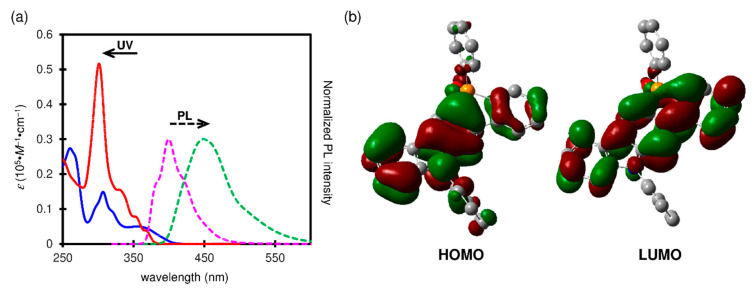
(**a**) UV–vis absorption and PL spectra of **8a** and **9** in CH_2_Cl_2;_ and (**b**) frontier molecular orbitals of **9**.

The longest absorption maximum of **9** (367 nm) was slightly red-shifted in comparison with that of **8a**, while the absorption edge was blue-shifted (Figure 8). In the PL spectrum, compound **9** showed largely blue-shifted emission maximum (402 nm) and smaller Φ compared to those of **8a**. The same trend was observed for the benzophosphoro[3,2-*b*]benzophosphole derivative **1** (λ_abs_ 395 nm; λ_em_ 480 nm; Φ 0.98) [11] and its higher homolog (λ_abs_ 383 nm; λ_em_ 420 nm; Φ 0.81) [25]. These differences in absorption and emission spectra between **9** and **8a** are qualitatively demonstrated by theoretical calculations. The calculated longest wavelength absorption of **9** (345 nm) is blue-shifted compared to that of **8a** (360 nm). Geometry optimization of the excited state [B3LYP/6-31+G(d)] also demonstrated a blue-shifted emission band of **9** (393 nm) compared to that of **8a** (461 nm). These experimental and theoretical results indicate that the separation of the pyrrole and the phosphole units by a benzene ring induces blue-shifted absorption and emission bands and smaller Stokes shifts.

## 3. Materials and Methods

### 3.1. General Procedures

All manipulations involving air- and/or moisture-sensitive compounds were carried out with the standard Schlenk technique under argon. Analytical thin-layer chromatography was performed on a glass plate coated with 0.25-mm 230–400-mesh silica gel containing a fluorescent indicator. Column chromatography was performed by using silica gel (spherical neutral, particle size: 63–210 μm). Most of reagents were purchased from commercial suppliers, such as Sigma-Aldrich Co. LLC (St. Louis, MO, USA), Tokyo Chemical Industry Co., Ltd. (Tokyo, Japan), and Kanto Chemical Co., Inc. (Tokyo, Japan), and used without further purification unless otherwise specified. Commercially available anhydrous solvents were used for air- and/or moisture-sensitive reactions. Compound **10** [31] and 3-bromo-2-(2-bromophenyl)carbazole [30] were prepared according to the literature.

NMR spectra were recorded in CDCl_3_ on a JEOL−ECA500 spectrometer (^1^H 500 MHz; ^13^C 126 MHz; ^31^P 202 MHz) or a JEOL−ECX400 spectrometer (^1^H 400 MHz; ^13^C 101 MHz; ^31^P 162 MHz) (JEOL Ltd., Tokyo, Japan). Chemical shifts are reported in ppm relative to the internal standard signal (0 ppm for Me_4_Si in CDCl_3_) for ^1^H and the deuterated solvent signal (77.16 ppm for CDCl_3_) for ^13^C. Data are presented as follows: chemical shift, multiplicity (s = singlet, d = doublet, dd = doublet of doublets, t = triplet, td = triplet of doublets, q = quartet, m = multiplet and/or multiple resonances), coupling constant in hertz (Hz), and signal area integration in natural numbers. Melting points were determined on an SRS OptiMelt melting point apparatus (Stanford Research Systems, Sunnyvale, CA, USA). High-resolution mass spectra were taken with Bruker Daltonics micrOTOF-QII mass spectrometer (Bruker Corporation, Billerica, MA, USA) using atmospheric pressure chemical ionization time-of-flight (APCI–TOF) method. UV–Vis absorption spectra were recorded on a JASCO V–650 spectrophotometer (JASCO Corporation, Tokyo, Japan). Photoluminescence spectra were recorded on a JASCO FP−6500 spectrofluorometer (JASCO Corporation, Tokyo, Japan). Absolute quantum yields were determined using absolute quantum yield measurement system with a JASCO ILF–533 integrating sphere (JASCO Corporation, Tokyo, Japan). The recycling preparative HPLC was performed with YMC-GPC T-2000 and T-4000 columns (chloroform as an eluent).

### 3.2. Synthesis

*2-[(2-Bromophenyl)ethynyl]-N-phenylaniline* (**10**). A Schlenk tube was charged with 2-iodo-*N*-phenylaniline (197 mg, 0.67 mmol), 1-bromo-2-ethynylbenzene (80 μL, 0.65 mmol), Pd(PPh_3_)_2_Cl_2_ (14 mg, 0.020 mmol), CuI (20 mg, 0.11 mmol), and triethylamine (8 mL). After degassed by three freeze-thaw-pump cycles, the resulting mixture was stirred at 70 °C for 18 h under argon atmosphere. Then, the reaction mixture was cooled to room temperature, and concentrated under reduced pressure to remove trimethylamine. The resulting crude residue was purified using silica-gel column chromatography (hexane as eluent, *R*_f_ = 0.20) to produce compound **10** as a yellow solid (220 mg, 97% yield): mp 81–83 °C; ^1^H NMR (500 MHz, CDCl_3_) *δ* 7.59 (dd, *J* = 8.0, 1.2 Hz, 1H), 7.55 (dd, *J* = 8.0, 1.7 Hz, 1H), 7.48 (dd, *J* = 7.7, 1.7 Hz, 1H), 7.33–7.25 (m, 4H), 7.23–7.18 (m, 3H), 7.14 (td, *J* = 7.7, 1.7 Hz, 1H), 7.55 (t, *J* = 7.5 Hz, 1H), 6.99 (brs, 1H), 6.80 (td, *J* = 7.5, 1.2 Hz, 1H); ^13^C NMR (126 MHz, CDCl_3_) *δ* 145.4, 141.5, 133.0, 132.5, 132.4, 130.1, 129.5, 129.4, 127.4, 125.4, 125.2, 122.6, 120.4, 119.0, 113.0, 109.4, 94.4, 90.9; HRMS−APCI^+^ (*m*/*z*) calcd for C_20_H_15_BrN ([M + H]^+^) 348.0382 (monoisotopic ion), found 348.0382.

*2-(2-Bromophenyl)-1-phenyl-1H-indole* (**11**). A 20-mL Schlenk tube was charged with compound **10** (0.63 mmol, 220 mg), Cu(OAc)_2_ (20 mol%, 23 mg), decanoic acid (27 mol%, 30 mg), and toluene (5 mL). The resulting mixture was refluxed at 125 °C under air for 18 h. Then, the reaction mixture was cooled to room temperature and diluted with CHCl_3_. The organic layer was washed with H_2_O, dried over anhydrous Na_2_SO_4_, filtered, and concentrated under reduced pressure. The resulting crude residue was purified using silica-gel column chromatography (hexane as an eluent, *R*_f_ = 0.16) to produce compound **11** as a yellow oil (217 mg, 98% yield). The ^1^H NMR data of the obtained product were identical to that published previously [22].

*3-Bromo-2-(2-bromophenyl)-1-phenyl-1H-indole* (**12**). A Schlenk tube was charged with compound **11** (1.32 g, 3.8 mmol) and anhydrous THF (40 mL) under argon atmosphere. To the mixture was added *N*-bromosuccinimide (673 mg, 3.8 mmol) at 0 °C. Then, the resulting mixture was warmed to room temperature and stirred for 21 h. The reaction mixture was diluted with CHCl_3_ and washed with saturated aqueous Na_2_S_2_O_3_. The organic layer was dried over anhydrous Na_2_SO_4_, filtered, and concentrated under reduced pressure. The resulting crude residue was purified using silica-gel column chromatography (hexane as eluent, *R*_f_ = 0.30) to produce compound **12** as a colorless solid (1.50 g, 93% yield): mp 142–146 °C; ^1^H NMR (500 MHz, CDCl_3_) *δ* 7.69 (dd, *J* = 6.9, 1.7 Hz, 1H), 7.54 (d, *J* = 8.0, 1H), 7.32–7.22 (m, 10H), 7.16 (ddd, *J* = 8.0, 6.9, 2.3 Hz, 1H),; ^13^C NMR (126 MHz, CDCl_3_) *δ* 137.5, 136.9, 136.8, 133.4, 132.8, 132.4, 130.6, 129.2, 127.7, 127.6, 127.14, 127.08, 125.6, 123,8, 121.4, 119.6, 111.0, 94.3; HRMS−APCI^+^ (*m*/*z*) calcd for C_20_H_14_Br_2_N ([M + H]^+^) 425.9488 (monoisotopic ion), found 425.9489.

*5,10-Diphenyl-5H-phosphindolo[3,2-b]indole 10-oxide* (**8a**). A 30-mL Schlenk tube was charged with **12** (85 mg, 0.20 mmol), Et_2_O (5 mL), and *N*,*N*,*N′*,*N′*-tetramethylethylenediamine (TMEDA, 0.12 mL, 0.80 mmol) under argon atmosphere. The resulting mixture was cooled to 0 °C, and *n*-butyllithium (0.22 mL of 2.07 M solution in hexane, 0.46 mmol) was added dropwise to the mixture at 0 °C. After stirring at the same temperature for 15 min, PhPCl_2_ (50 μL, 0.37 mmol) was added in one portion to the reaction mixture. The resulting mixture was allowed to warm quickly to 25 °C, stirred at the same temperature for 10 min, and concentrated under reduced pressure.

The resulting crude residue, which would contain compound **5**, was dissolved in CH_2_Cl_2_ (5 mL) under argon atmosphere, and H_2_O_2_ (0.50 mL, 35% aqueous solution) was added in one portion to the solution. After the resulting mixture was stirred at 25 °C for 25 min, saturated aqueous Na_2_S_2_O_3_ was slowly added to the reaction mixture. The organic layer was separated, and the aqueous layer was extracted with EtOAc (10 mL × 3). The combined organic layers were washed with brine, dried over Na_2_SO_4_, filtered, and concentrated under reduced pressure. The resulting crude residue was purified using silica-gel column chromatography [EtOAc/haxane (3/1) as eluent, *R*_f_ = 0.45] to produce the title compound as a colorless solid (51 mg, 65% yield): mp 215–220 °C; ^1^H NMR (400 MHz, CDCl_3_) *δ* 7.89 (dd, *J* = 8.0, 1.4 Hz, 1H), 7.85 (dd, *J* = 7.8, 1.8 Hz, 1H), 7.69–7.63 (m, 5H), 7.55–7.47 (m, 3H), 7.43–7.38 (m, 2H), 7.23–7.08 (m, 5H), 6.63 (dd, *J* = 7.3, 3.2 Hz, 1H); ^13^C NMR (101 MHz, CDCl_3_) *δ* 149.7 (d, *J* = 34.5 Hz), 143.7 (d, *J* = 9.6 Hz), 140.1 (d, *J* = 106.4 Hz), 136.7, 134.5 (d, *J* = 14.4 Hz), 132.1 (d, *J* = 2.9 Hz), 132.0, 131.4, (d, *J* = 110.2 Hz), 131.2 (d, *J* = 10.5 Hz), 130.3, 130.04, 130.00 (d, *J* = 8.6 Hz), 129.7, 128.95 (d, *J* = 11.5 Hz), 128.8 (d, *J* = 12.5 Hz), 128.4, 128.1, 125.6 (d, *J* = 8.6 Hz), 123.9, 122.8, 120.9, 120.7 (d, *J* = 8.6 Hz), 111.6, 108.1 (d, *J* = 128.4 Hz); ^31^P NMR (162 MHz, CDCl_3_) *δ* 22.4; HRMS–APCI^+^ (*m*/*z*) calcd for C_26_H_19_NOP^+^ ([M + H]^+^) 392.1199, found 392.1193.

*10-(4-Methoxyphenyl)-5-phenyl-5H-phosphindolo[3,2-b]indole 10-oxide* (**8b**). A 30-mL Schlenk tube was charged with **12** (128 mg, 0.30 mmol), Et_2_O (6.0 mL), and *N,N,N′,N′*-tetramethylethylenediammine (TMEDA, 0.18 mL, 1.2 mmol) under argon atmosphere. The mixture was cooled to −78 °C, and *n*-butyllithium (0.30 mL of 2.3 M solution in hexane, 0.67 mmol) was added dropwise to the mixture. After stirred at the same temperature for 1 h, (Et_2_N)PCl_2_ (58 μL, 0.39 mmol) was added dropwise to the mixture. The resulting mixture was stirred at the same temperature for 30 min and allowed to warm quickly to 25 °C. After stirring for 17 h, the reaction mixture was passed through a short pad of neutral alumina with Et_2_O, and the neutral alumina was rinsed with hexane. The filtrate was concentrated to give the crude product of aminophosphole intermediate (41 mg), which was used for the next step without further purification.

A 50-mL Schlenk tube was charged with the crude product of aminophosphole intermediate, toluene (0.80 mL), and methanol (0.60 mL) under argon atmosphere. The resulting mixture was stirred at 65 °C for 4 h. Concentration of the mixture afforded the crude product of methoxyphosphole intermediate **13** as a yellow solid (42 mg), which was used for the next step without further purification.

A 30-mL Schlenk tube was charged with 1-bromo-4-methoxybenzene (32 μL, 0.26 mmol), THF (0.60 mL), and TMEDA (75 μL, 0.51 mmol) under argon atmosphere. The mixture was cooled to −78 °C, and *n*-butyllithium (0.12 mL of 2.3 M solution in hexane, 0.27 mmol) was added dropwise to the mixture. After stirring at the same temperature for 1 h, the resulting mixture was added to the THF solution (0.30 mL) of the crude product of methoxyphosphole intermediate **13** (42 mg, 0.13 mmol) dropwise at −78 °C. After stirring at the same temperature for 30 min, the reaction mixture was allowed to warm to 50 °C and stirred for 14 h. The reaction mixture was concentrated under reduced pressure, and the residue was dissolved with Et_2_O. The resulting solution was passed through a short pad of neutral alumina with Et_2_O. The filtrate was concentrated, and the residue was dissolved with CH_2_Cl_2_ (1.3 mL) under argon atmosphere. H_2_O_2_ (2 mL, 35% aqueous solution) was added to the resulting solution, and the resulting mixture was stirred at 25 °C for 1.5 h. After the reaction mixture was diluted with H_2_O, the resulting mixture was extracted with CH_2_Cl_2_ twice. The combined organic layer was washed with saturated aqueous Na_2_S_2_O_3_, dried over Na_2_SO_4_, filtered, and concentrated under reduced pressure. The resulting reside was purified using silica-gel column chromatography (EtOAc as eluent, *R*_f_ = 0.23) to produce **8b** as a colorless solid (21 mg, 17% yield): mp 103–115 °C; 7.81 (dd, *J* = 6.9, 1.8 Hz, 1H), 7.78 (dd, *J* = 6.9, 1.8 Hz, 1H), 7.69–7.61 (m, 5H), 7.55–7.53 (m, 2H), 7.24–7.09 (m, 5H), 6.94 (d, *J* = 8.7 Hz, 1H), 6.93 (d, *J* = 8.7 Hz, 1H), 6.61 (dd, *J* = 7.8, 3.2 Hz, 1H), 3.81 (s, 3H); ^13^C NMR (101 MHz, CDCl_3_) *δ* 162.9 (d, *J* = 2.9 Hz), 149.7 (d, *J* = 34.5 Hz), 143.8 (d, *J* = 9.6 Hz), 140.7 (d, *J* = 106.4 Hz), 136.9, 134.5 (d, *J* = 14.4 Hz), 133.2 (d, *J* = 12.5 Hz), 131.9, 130.3, 130.1, 129.9 (d, *J* = 9.6 Hz), 129.7, 129.0 (d, *J* = 11.5 Hz), 128.5, 128.2, 125.7 (d, *J* = 8.6 Hz), 123.8, 122.9, 122.1 (d, *J* = 116.9 Hz), 121.0, 120.7 (d, *J* = 8.6 Hz); 114.6 (d, *J* = 13.4 Hz); 111.6, 108.4 (d, *J* = 128.4 Hz), 55.4; ^31^P NMR (162 MHz, CDCl_3_) *δ* 22.4; HRMS–APCI^+^ (*m*/*z*) calcd for C_27_H_21_NO_2_P^+^ ([M + H]^+^) 422.1304, found 422.1311.

*10-[(N,N-Dimethylamino)phenyl]-5-phenyl-5H-phosphindolo[3,2-b]indole 10-oxide* (**8c**). The crude residue was obtained by using **12** (128 mg, 0.30 mmol) and 4-bromo-*N*,*N*-dimethylaniline (53 mg, 0.26 mmol). The resulting residue was purified using silica-gel column chromatography (EtOAc as eluent, *R*_f_ = 0.22) as a pale yellow solid (6 mg, 5% yield): 7.73–7.61 (m, 7H), 7.54 (dd, *J* = 7.6, 2.0 Hz, 2H), 7.22–7.08 (m, 5H), 6.683 (d, *J* = 9.2 Hz, 1H), 6.677 (d, *J* = 9.2 Hz, 1H), 6.59 (dd, *J* = 7.8, 3.2 Hz, 1H), 2.98 (s, 6H); ^13^C NMR (101 MHz, CDCl_3_) *δ* 153.0 (d, *J* = 1.9 Hz), 149.6 (d, *J* = 33.6 Hz), 143.7 (d, *J* = 9.6 Hz), 141.3 (d, *J* = 106.4 Hz), 137.1, 134.3 (d, *J* = 14.4 Hz), 132.7 (d, *J* = 12.5 Hz), 131.5, 130.3, 130.0, 129.7 (d, *J* = 8.6 Hz), 129.6, 128.8 (d, *J* = 11.5 Hz), 128.6, 128.2, 125.8 (d, *J* = 8.6 Hz), 123.7, 122.7, 121.1, 120.6 (d, *J* = 8.6 Hz); 115.0 (d, *J* = 121.7 Hz), 111.8 (d, *J* = 14.4 Hz); 111.5, 108.9 (d, *J* = 127.5 Hz), 40.1; ^31^P NMR (162 MHz, CDCl_3_) *δ* 23.4; HRMS–APCI^+^ (*m*/*z*) calcd for C_28_H_24_N_2_OP^+^ ([M + H]^+^) 435.1621, found 435.1627.

*10-[4-(Trifluoromethyl)phenyl]-5-phenyl-5H-phosphindolo[3,2-b]indole 10-oxide* (**8d**). The crude residue was obtained by using **12** (171 mg, 0.40 mmol) and 1-bromo-4-(trifluoromethyl)benzene (36 μL, 0.26 mmol). The resulting residue was purified using silica-gel column chromatography [EtOAc/hexane (5/1) as an eluent, *R*_f_ = 0.50] as a pale yellow solid (37 mg, 20% yield): ^1^H NMR (400 MHz, CDCl_3_) *δ* 8.0 (d, *J* = 8.2 Hz, 1H), 7.97 (d, *J* = 8.2 Hz, 1H), 7.69–7.61 (m, 7H), 7.56–7.54 (m, 2H), 7.28–7.19 (m, 4H), 7.13–7.11 (m, 1H), 6.66 (dd, *J* = 7.1, 3.0 Hz, 2H); ^13^C NMR (101 MHz, CDCl_3_) *δ* 150.0 (d, *J* = 34.5 Hz), 143.8 (d, *J* = 10.5 Hz), 139.2 (d, *J* = 107.4 Hz), 136.6, 136.2 (d, *J* = 107.4 Hz), 134.6 (d, *J* = 15.3 Hz), 133.9 (qd, *J* = 32.6, 2.9 Hz), 132.5, 131.8 (d, *J* = 11.5 Hz), 130.4, 130.21 (d, *J* = 7.7 Hz), 130.17, 129.9, 129.3 (d, *J* = 11.5 Hz), 128.4, 128.1, 125.8 (dq, *J* = 13.4, 3.8 Hz), 125.4 (d, 8.6 Hz), 124.2, 123.7 (q, *J* = 272.2 Hz), 123.2, 121.0 (d, *J* = 9.6 Hz), 120.8, 111.8, 107.3 (d, *J* = 130.4 Hz); ^31^P NMR (162 MHz, CDCl_3_) *δ* 20.7; HRMS–APCI^+^ (*m*/*z*) calcd for C_27_H_18_F_3_NOP^+^ ([M + H]^+^) 460.1073, found 460.1078.

*3-Bromo-2-(2-bromophenyl)-9-phenyl-9H-carbazole* (**14**). A 30-mL Schlenk tube was charged with 3-bromo-2-(2-bromophenyl)-9*H*-carbazole (66 mg, 0.17 mmol), KO*^t^*Bu (44 mg, 0.39 mmol), and toluene (2 mL) under argon atmosphere. After stirring at 50 °C for 10 min, diphenyliodonium trifluoromethansulfonate (86 mg, 0.20 mmol) was added to the mixture. The resulting mixture was stirred at the same temperature for 28 h, and water was added to the reaction mixture. The resulting mixture was extracted with EtOAc (three times), and the combined organic layers were dried over Na_2_SO_4_, filtered, and concentrated under reduced pressure. The resulting crude reside was purified using silica-gel column chromatography [hexane/CH_2_Cl_2_ (10/1) as an eluent, *R*_f_ = 0.38] to give **14** as a colorless solid (57 mg, 72% yield): mp 80–90 °C; ^1^H NMR (400 MHz, CDCl_3_) *δ* 8.41 (s, 1H), 8.11 (dd, *J* = 7.8, 0.9 Hz, 1H), 7.65 (dd, *J* = 8.0, 0.9 Hz, 1H), 7.58–7.52 (m, 4H), 7.45–7.38 (m, 3H), 7.36–7.28 (m, 3H), 7.25–7.20 (m, 2H); ^13^C NMR (101 MHz, CDCl_3_) *δ* 143.0, 141.7, 139.8, 139.2, 137.3, 132.6, 131.5, 130.2, 129.4, 127.9, 127.15, 127.12, 126.9, 124.8, 124.2, 124.0, 122.2, 120.7, 120.6, 114.1, 112.0, 110.2; HRMS–APCI^+^ (*m*/*z*) calcd for C_24_H_16_Br_2_N^+^ ([M + H]^+^) 475.9644 (monoisotopic ion), found 475.9649.

*5,11-Diphenyl-5H-phosphindolo[3,2-b]carbazole 11-oxide* (**9**). A 50-mL Schlenk tube was charged with **14** (74 mg, 0.16 mmol), Et_2_O (4 mL), and TMEDA (95 μL, 0.64 mmol) under argon atmosphere. The mixture was cooled to −78 °C, and *n*-butyllithium (0.13 mL of 2.7 M solution in hexane, 0.34 mmol) was added dropwise to the mixture. After stirring at the same temperature for 2 h, PhPCl_2_ (27 μL, 0.20 mmol) was added in one portion to the reaction mixture. After stirring at the same temperature for 30 min, the reaction mixture was allowed to warm quickly to 25 °C and stirred for 18 h. The reaction mixture was concentrated under reduced pressure, and the resulting crude residue, which would contain trivalent phosphole intermediate, was dissolved with CH_2_Cl_2_ (8 mL) under argon atmosphere. H_2_O_2_ (1.0 mL, 35% aqueous solution) was added to the resulting solution, and the resulting mixture was stirred at 25 °C for 1 h. After the reaction was quenched with saturated aqueous Na_2_S_2_O_3_, the resulting mixture was extracted with CH_2_Cl_2_ (three times). The combined organic layers were dried over Na_2_SO_4_, filtered, and concentrated under reduced pressure. The resulting crude residue was purified using silica-gel column chromatography [EtOAc/hexane (2/1) as eluent, *R*_f_ = 0.30] and recycling preparative HPLC (CHCl_3_ as an eluent) to produce the title compound as a colorless solid (15 mg, 17% yield): ^1^H NMR (400 MHz, CDCl_3_) *δ* 8.47 (d, *J* = 9.6 Hz, 1H), 8.06 (d, *J* = 7.3 Hz, 1H), 7.79–7.67 (m, 8H), 7.61–7.47 (m, 5H), 7.44–7.28 (m, 6H); ^13^C NMR (101 MHz, CDCl_3_) *δ* 144.4, 142.5 (d, *J* = 21.1 Hz), 142.2, 140.1 (d, *J* = 24.0 Hz), 136.9, 134.3 (d, *J* = 106.4 Hz), 133.2 (d, *J* = 1.9 Hz), 132.1 (d, *J* = 2.9 Hz), 132.0 (d, *J* = 104.5 Hz), 131.4 (d, *J* = 11.5 Hz), 130.4, 129.9 (d, *J* = 9.6 Hz), 129.3 (d, *J* = 10.5 Hz), 128.8 (d, *J* = 12.5 Hz), 128.5, 127.5, 127.0, 124.7 (d, *J* = 13.4 Hz), 123.9 (d, *J* = 113.1 Hz), 123.1 (d, *J* = 10.5 Hz), 123.0, 121.25, 121.19 (d, *J* = 11.5 Hz), 120.6, 110.3, 102.7 (d, *J* = 11.5 Hz); ^31^P NMR (202 MHz, CDCl_3_) *δ* 33.5; HRMS–APCI^+^ (*m*/*z*) calcd for C_30_H_21_NOP^+^ ([M + H]^+^) 442.1355, found 442.1361.

### 3.3. X-ray Crystallography

For X-ray crystallographic analyses, suitable single crystals were selected under ambient conditions, mounted using a nylon loop filled with paraffin oil, and transferred to the goniometer of a RIGAKU R-AXIS RAPID diffractometer with a graphite-monochromated Cu−Kα irradiation (λ = 1.54187 Å) (Tokyo, Japan). The structures were solved using a direct method (SIR 2008 [32]) and refined by full-matrix least-squares techniques against *F*2 (SHELXL-2014 [33,34]). The intensities were corrected for Lorentz and polarization effects. All non-hydrogen atoms were refined anisotropically. Hydrogen atoms were placed using AFIX instructions.

Crystal Data for **8a**: Formula: C_26_H_18_NOP (*M* = 391.38 g/mol): monoclinic, space group *P*2_1_/*c* (No. 14), *a* = 9.0262(2) Å, *b* = 10.9540(2) Å, *c* = 20.2813(4) Å, *β* = 100.0930(10)°, *V* = 1974.24(7) Å^3^, *Z* = 4, *T* = 193(2) K, *μ*(CuKα) = 1.54187 mm^−1^, *D*_calc_ = 1.317 g/cm^3^, 35030 reflections measured (4.429° ≤ *θ* ≤ 68.229°), 3597 unique (*R*_int_ = 0.0354; *R*_sigma_ = 0.0198), which were used in all calculations. The final *R*_1_ was 0.0412 (*I* > 2σ(*I*)) and w*R*_2_ was 0.1191 (all data).

Crystal Data for **9**: Formula: C_30_H_20_NOP·H_2_O (*M* = 459.45 g/mol): monoclinic, space group *C2*/*c* (No. 15), *a* = 36.7954(7) Å, *b* = 6.48420(12) Å, *c* = 19.5726(4) Å, *β* = 102.4869(8)°, *V* = 4559.33(15) Å^3^, *Z* = 8, *T* = 193(2) K, *μ*(CuKα) = 1.54187 mm^−1^, *D*_calc_ = 1.339 g/cm^3^, 38,357 reflections measured (4.628° ≤ *θ* ≤ 68.219°), 4170 unique (*R*_int_ = 0.0292; *R*_sigma_ = 0.0174), which were used in all calculations. The final *R*_1_ was 0.0502 (*I* > 2σ(*I*)) and w*R*_2_ was 0.1482 (all data).

CCDC 2311726 (**8a**) and 2311727 (**9**) contains the supplementary crystallographic data for this paper. The data can be obtained free of charge from The Cambridge Crystallographic Data Centre via www.ccdc.cam.ac.uk/structures.

### 3.4. Computational Studies

The DFT and TD–DFT calculations were performed using the Gaussian 16 [24] program at the B3LYP/6-31+G(d) level. The starting molecular models for DFT geometry optimizations were built and optimized with MMFF molecular mechanics using the Spartan ’08 package (Wavefunction, Inc., Irvine, CA, USA). Six singlet states were calculated in TD–DFT calculations. The visualization of the molecular orbitals was performed using GaussView 5.

## 4. Conclusions

In summary, we synthesized benzophospholo[3,2-*b*]indole derivatives (BPIs). The photophysical properties of the BPIs were revealed through UV–vis absorption and photoluminescence spectroscopies and theoretical calculations. The methoxy and trifluoromethyl groups on the *P*-aryl unit were found to have little impact on the absorption and photoluminescence properties, while the 4-dimethylamino group induces a slight red-shift in the absorption spectrum and a lower fluorescence quantum yield. These photophysical properties were well demonstrated by the theoretical calculations. The BPIs exhibit larger Stokes shifts compared with the analog compounds where the phosphole rings are directly fused with the other heteroles such as the silole, thiophene, and furan rings. Insertion of a benzene ring between the pyrrole and the phosphole units induces a blue-shift of the absorption edge and emission band.

## Figures and Tables

**Table 1:** Photophysical data of BPI **8**.

	λ_abs_ (nm) ^a^	λ_em_ (nm) ^b^	Stokes Shift (cm^−1^)	Φ ^c^
**8a**	355	449	5900	0.72
**8b**	355	449	5900	0.68
**8c**	---	448	---	0.50
**8d**	358	453	5860	0.68

^a^ In CHCl_3_. ^b^ In CHCl_3_. Excitation at 330 nm. ^c^ Absolute quantum yield determined using a calibrated integrating sphere system.

**Table 2:** Solvent effect on absorption and emission properties of **5**.

Solvent	*E*_T_(30) ^a^ (kcal·mol^−1^)	λ_abs_ (nm)	λ_em_ (nm) ^b^	Stokes Shift (cm^−1^)
C_6_H_6_	34.5	353	441	5650
CHCl_3_	39.1	355	449	5900
CH_2_Cl_2_	41.1	353	449	6060
CH_3_CN	46.0	349	452	6530
CH_3_OH	55.5	355	459	6380

^a^ Empirical parameters of solvent polarity [23]. ^b^ Excitation at 330 nm.

## Data Availability

The data presented in this study are available in Supplementary Material.

## Supplementary Materials

The following supporting information can be downloaded at: https://www.mdpi.com/article/10.3390/molecules29010038/s1, Figures S1−S21: ^1^H and ^13^C NMR spectra for **8a**–**8d**, **9**, **10**, **12**, and **14**; Tables S1 and S2: Crystallographic data for **8a** and **9**; Figure S22: UV/Vis absorption spectra of **8a** in various solvents; Figures S23−S27: Molecular orbitals for **8a**–**8d** and **9**; Tables S3–S7: Coordinates and absolute energy of the optimized structures for **8a**–**8d** and **9**; Table S8: The selected absorption of **8a**–**8d** and **9** calculated by TD−DFT method.
