# Supplementary material for: The Synthesis and Properties of Ladder-Type π-Conjugated Compounds with Pyrrole and Phosphole Rings
Source: Molecules. 2023 Dec 20;29(1):38. doi: 10.3390/molecules29010038 (PMC10779499; doi:10.3390/molecules29010038)
Supplement: Supplementary file 1 [file molecules-29-00038-s001.zip › SI.pdf]

Supporting Information

Synthesis and Properties of Ladder-type π-Conjugated Compounds with Pyrrole and Phosphole Rings

Minh Anh Truong,[1] Suzuho Morishita,[1] Keiichi Noguchi,[2] Koji Nakano[*,1]

[1]Department of Organic and Polymer Materials Chemistry, and [2]Instrumentation Analysis Center, Tokyo University of Agriculture and Technology, 2-24-16 Naka-cho, Koganei, Tokyo 184-8588, Japan

e-mail: k_nakano@cc.tuat.ac.jp

Table of Contents

^{1}H and ^{13}C Spectra of Compounds **8a–8d**, **9**, **10**, **12**, and **14** S2−S12

X-ray Analysis S13−S14

UV/Vis Absorption Spectra of **8a** in Various Solvents S15

DFT and TD-DFT Calculation Results S16−S28

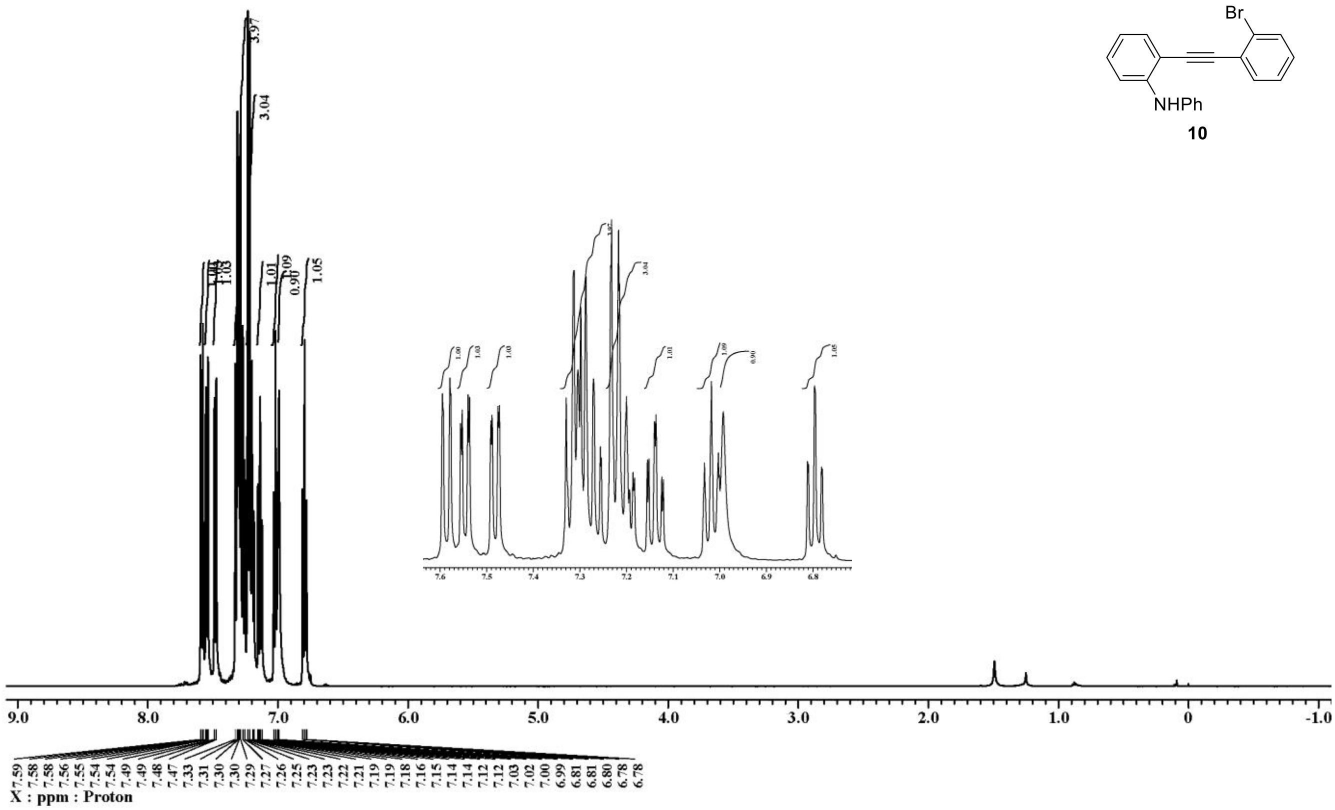

Figure S1. ^{1}H NMR spectrum of **10** (500 MHz, $CDCl_3$).

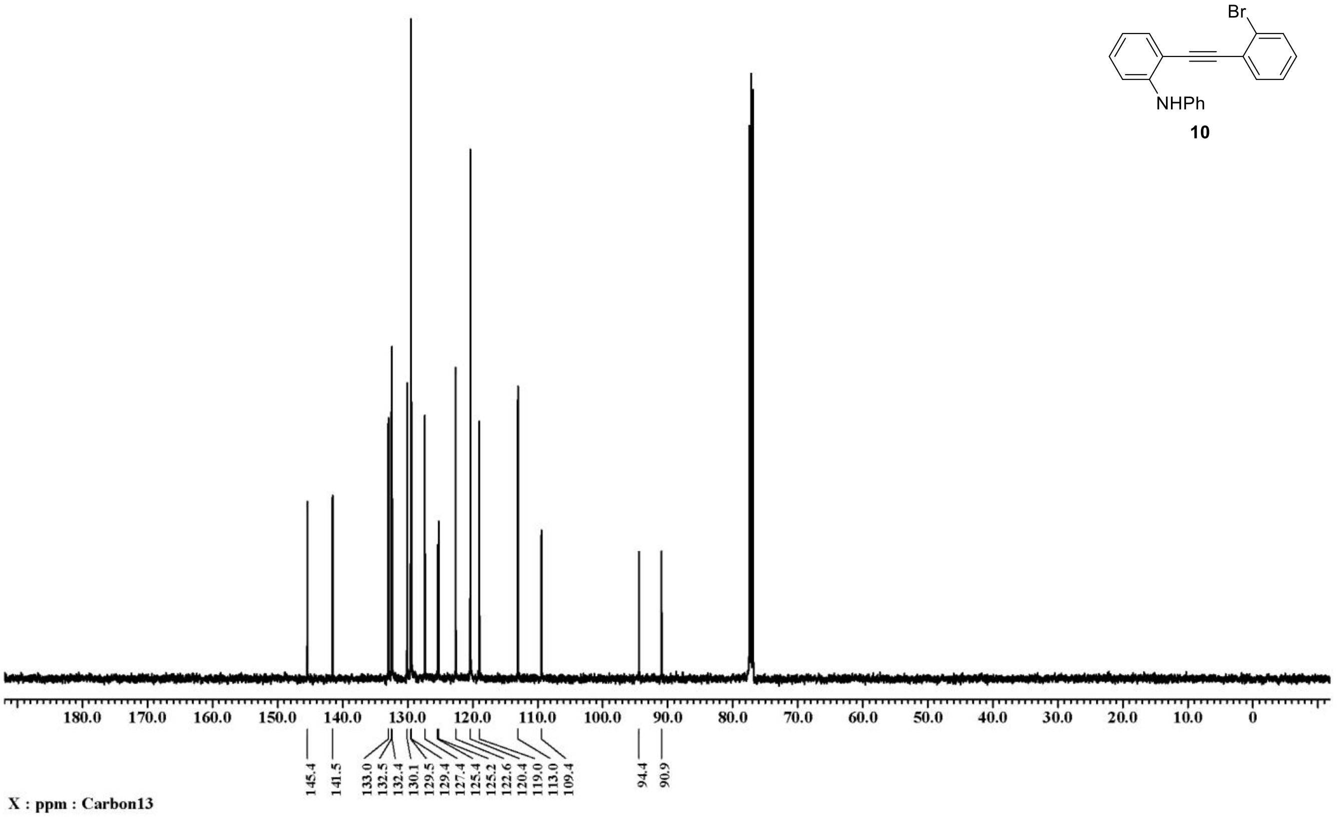

Figure S2. ^{13}C NMR spectrum of **10** (126 MHz, $CDCl_3$).

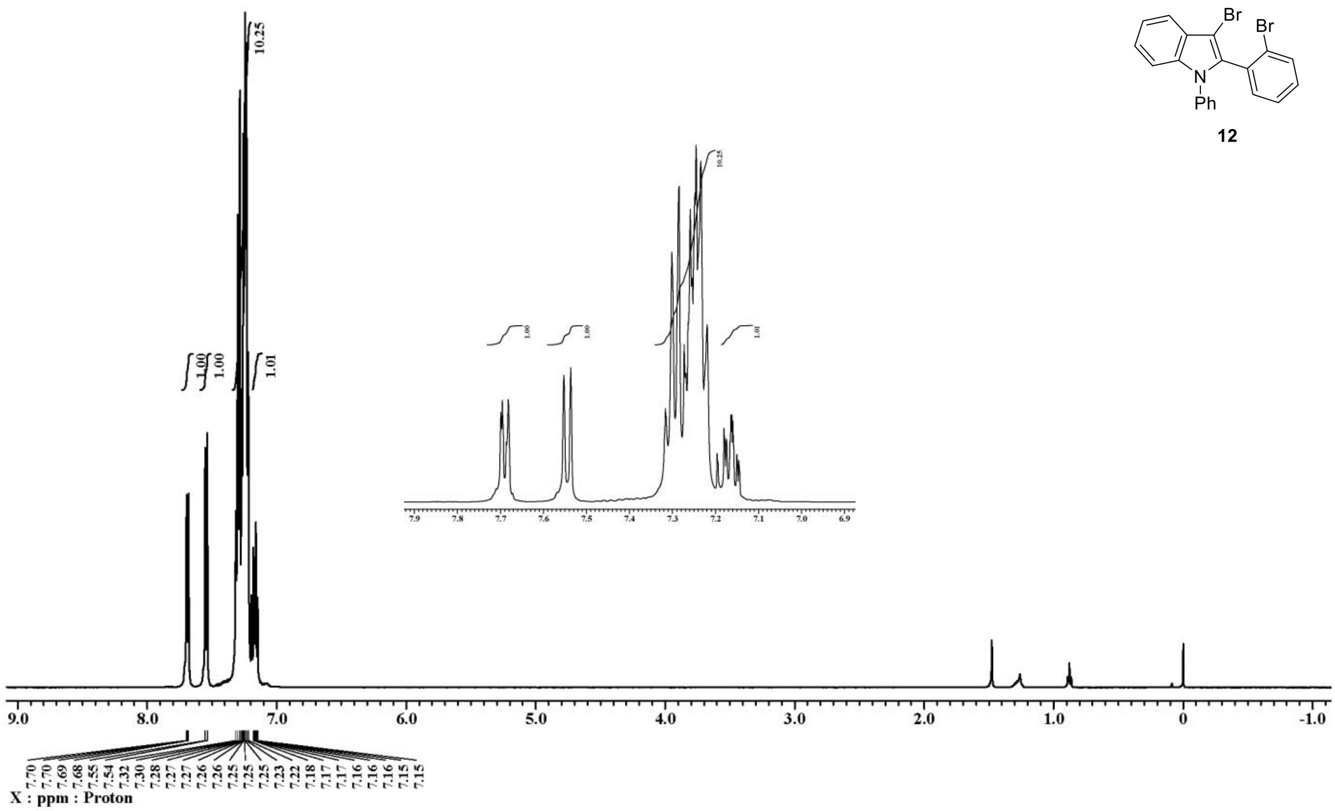

Figure S3. ^{1}H NMR spectrum of **12** (500 MHz, $CDCl_3$).

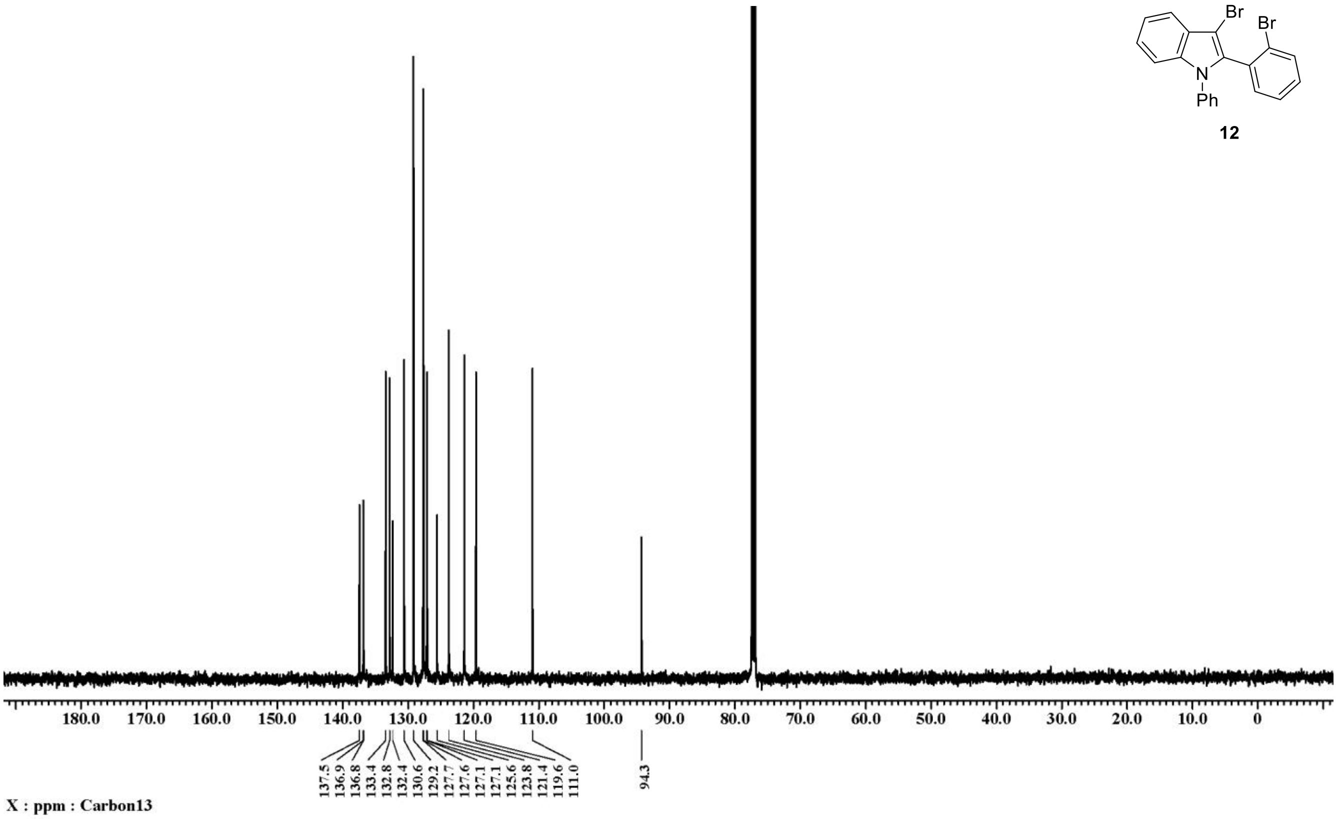

Figure S4. ^{13}C NMR spectrum of **12** (126 MHz, $CDCl_3$).

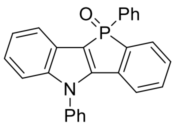

8a

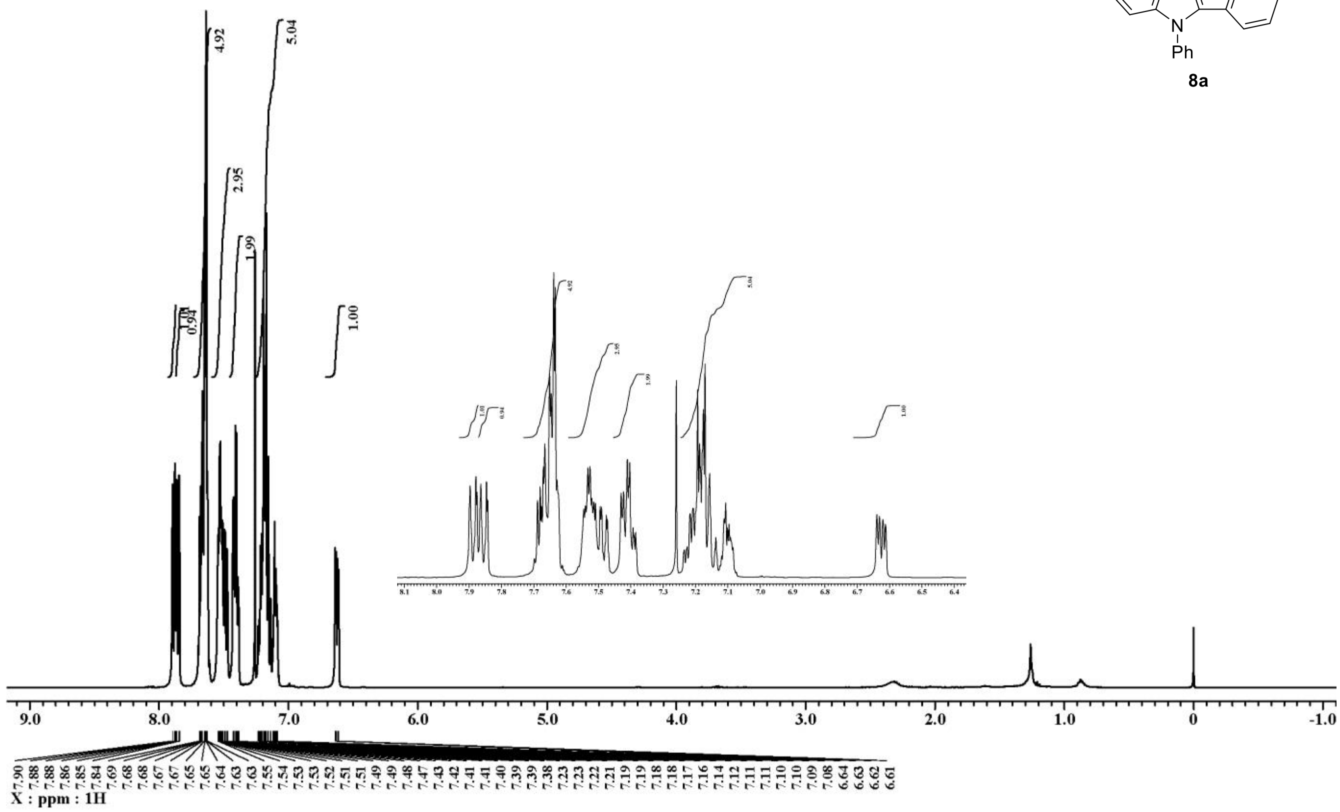

Figure S5. ^{1}H NMR spectrum of **8a** (400 MHz, $CDCl_3$).

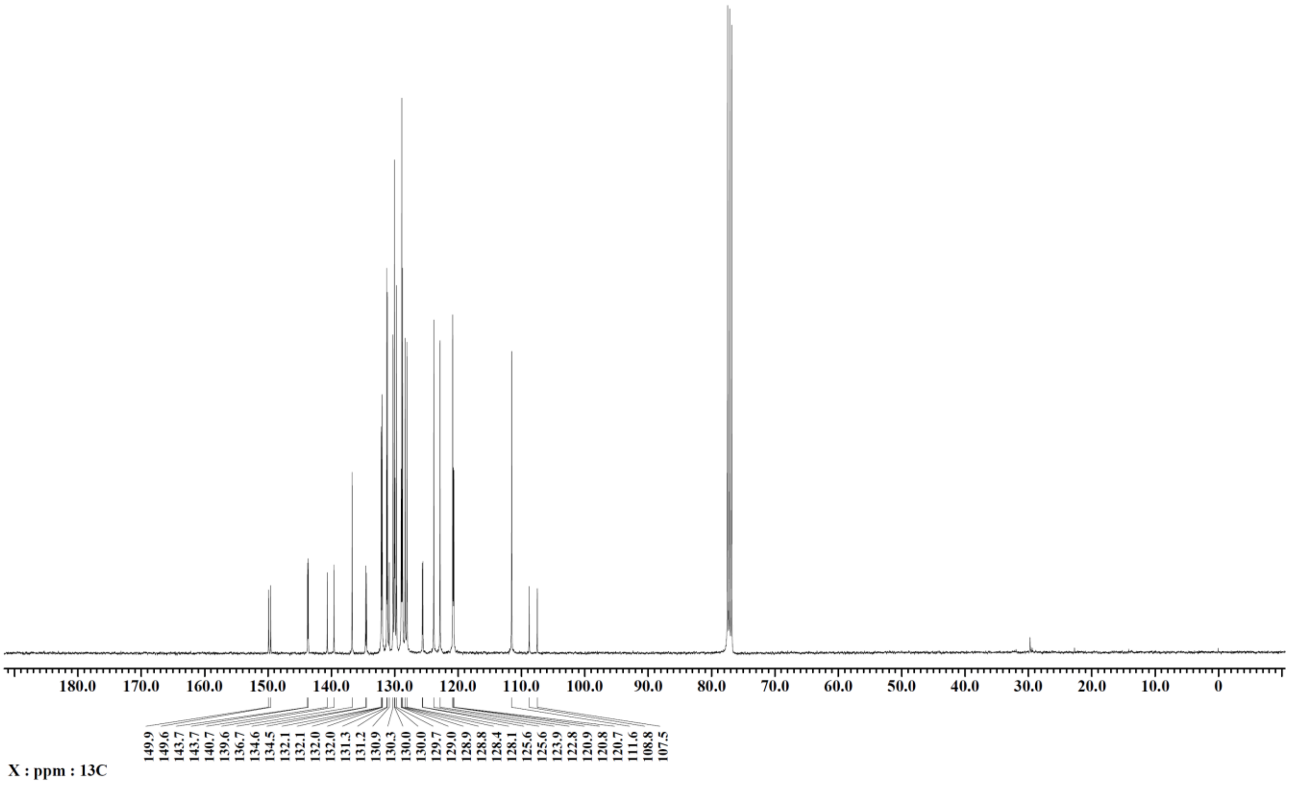

Figure S6. ^{13}C NMR spectrum of **8a** (101 MHz, $CDCl_3$).

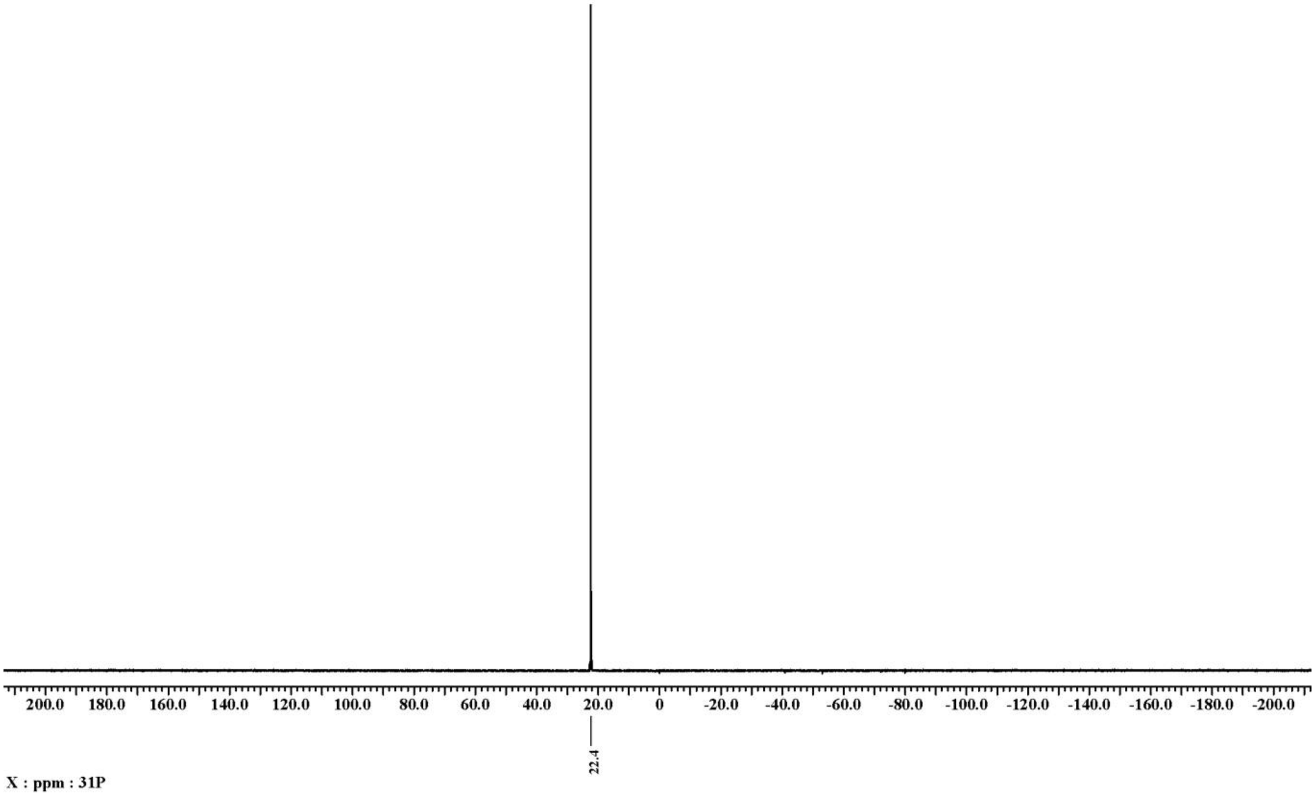

Figure S7. ^{31}P NMR spectrum of **8a** (162 MHz, $CDCl_3$).

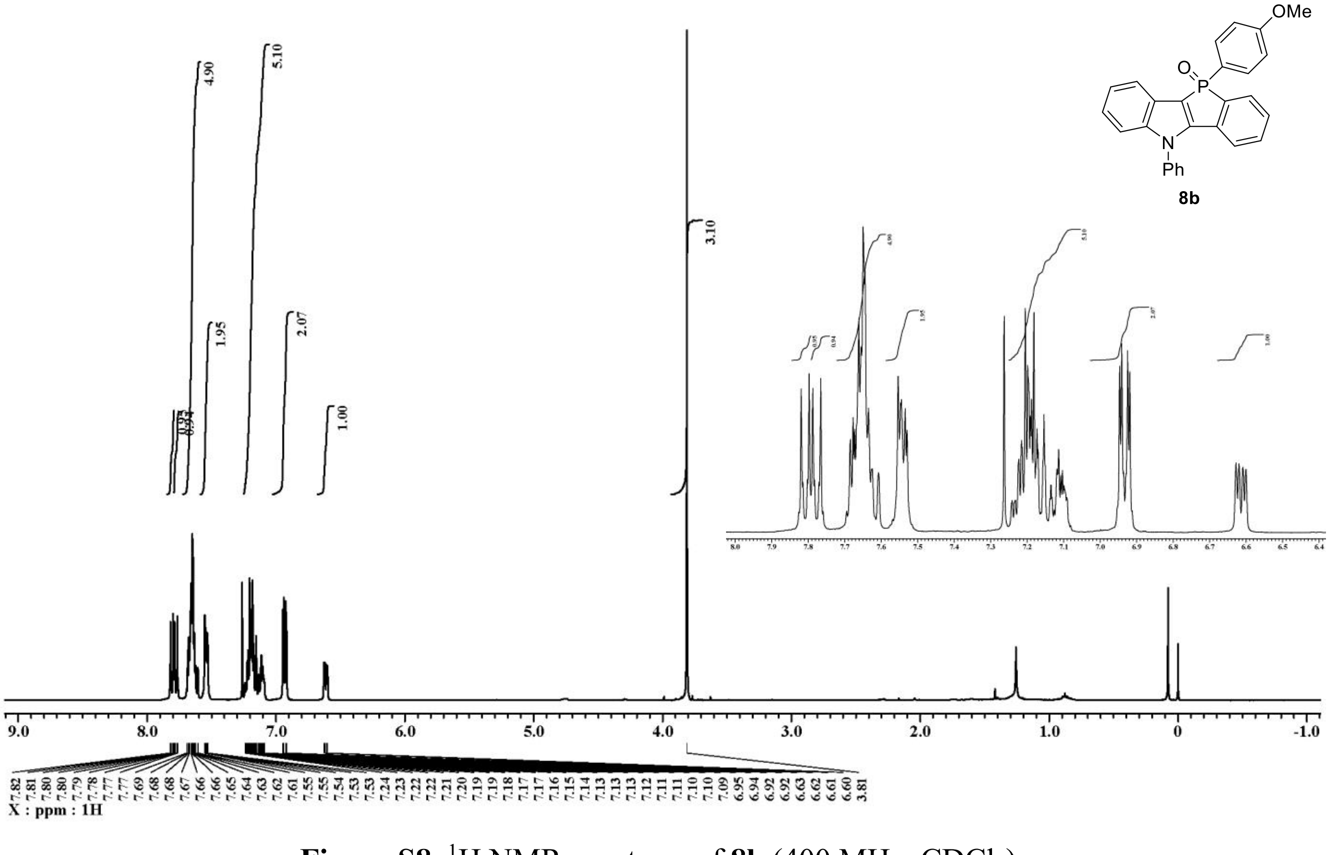

Figure S8. ^{1}H NMR spectrum of **8b** (400 MHz, $CDCl_3$).

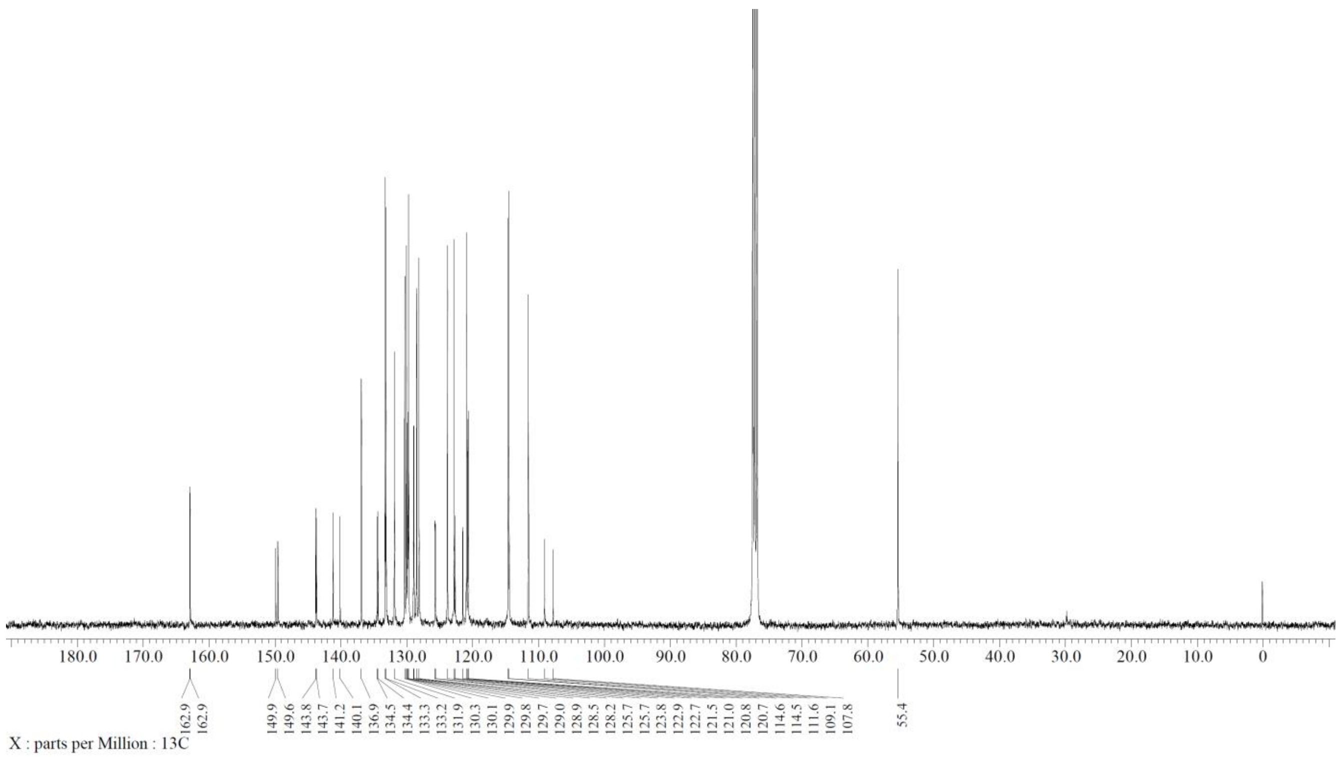

Figure S9. ^{13}C NMR spectrum of **8b** (101 MHz, $CDCl_3$).

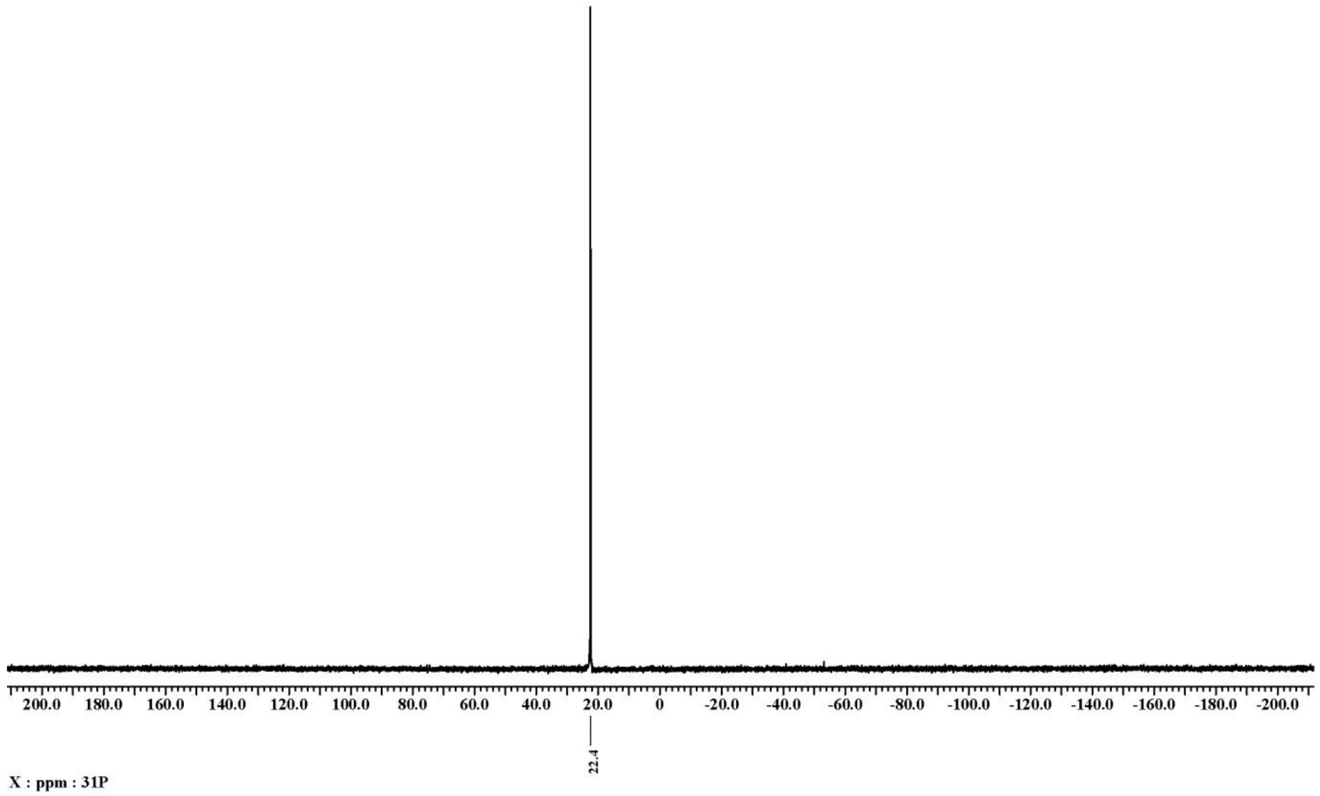

Figure S10. ^{31}P NMR spectrum of **8b** (162 MHz, $CDCl_3$).

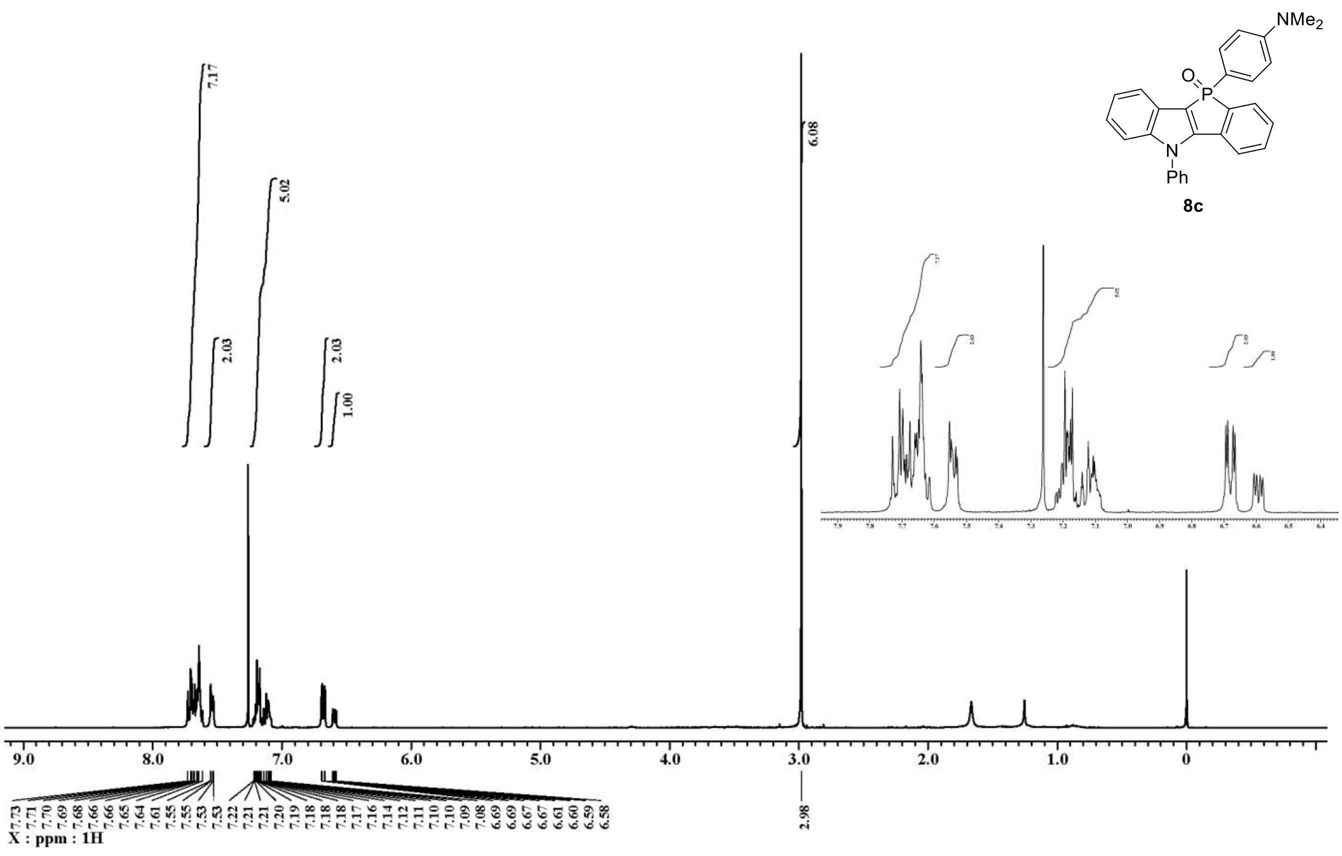

Figure S11. ^{1}H NMR spectrum of **8c** (400 MHz, $CDCl_3$).

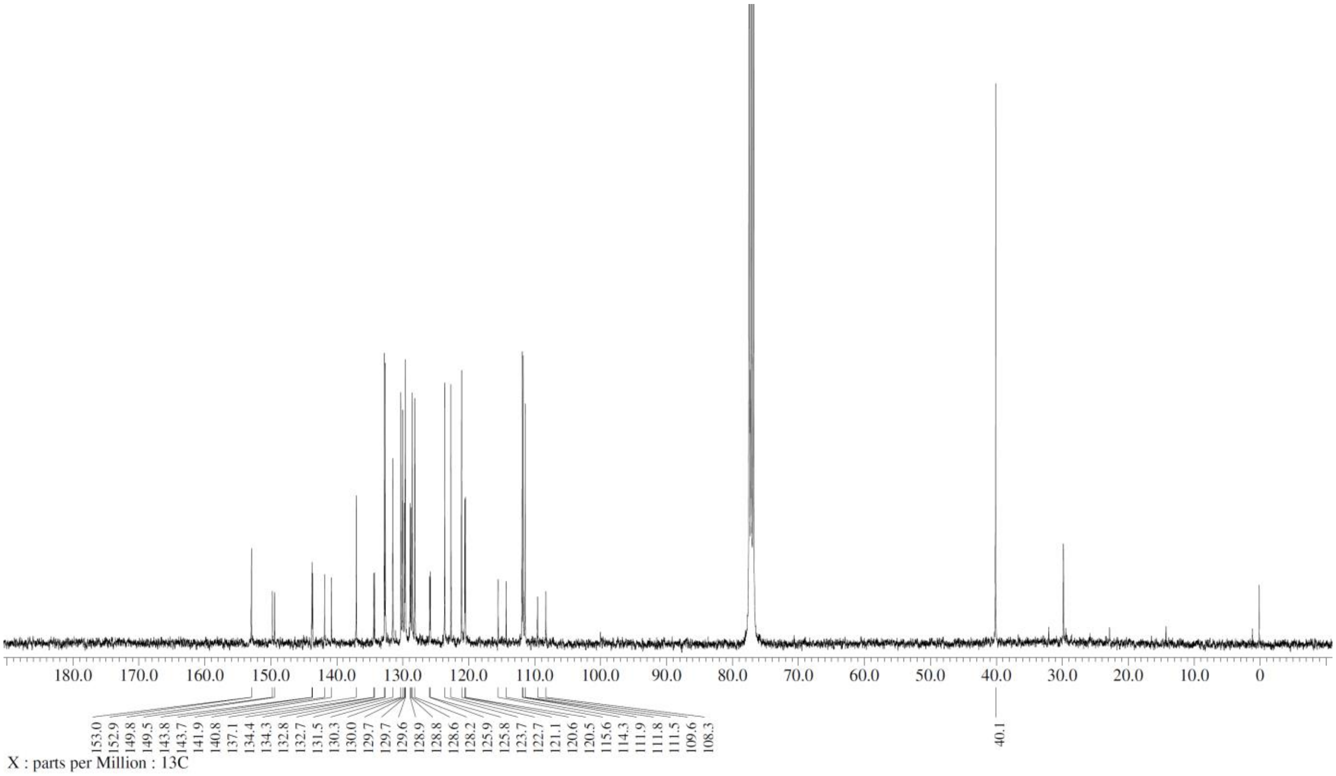

Figure S12. ^{13}C NMR spectrum of **8c** (101 MHz, $CDCl_3$).

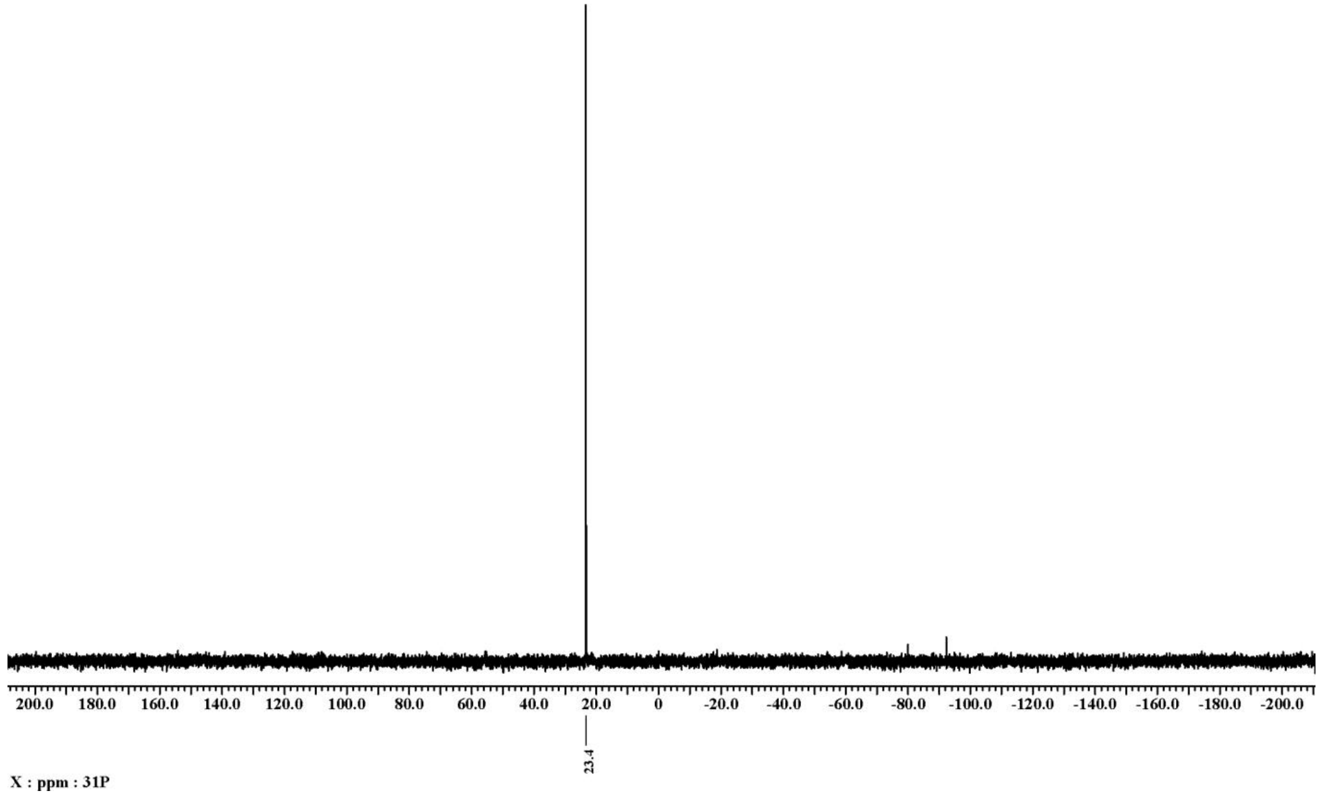

Figure S13. ^{31}P NMR spectrum of **8c** (162 MHz, $CDCl_3$).

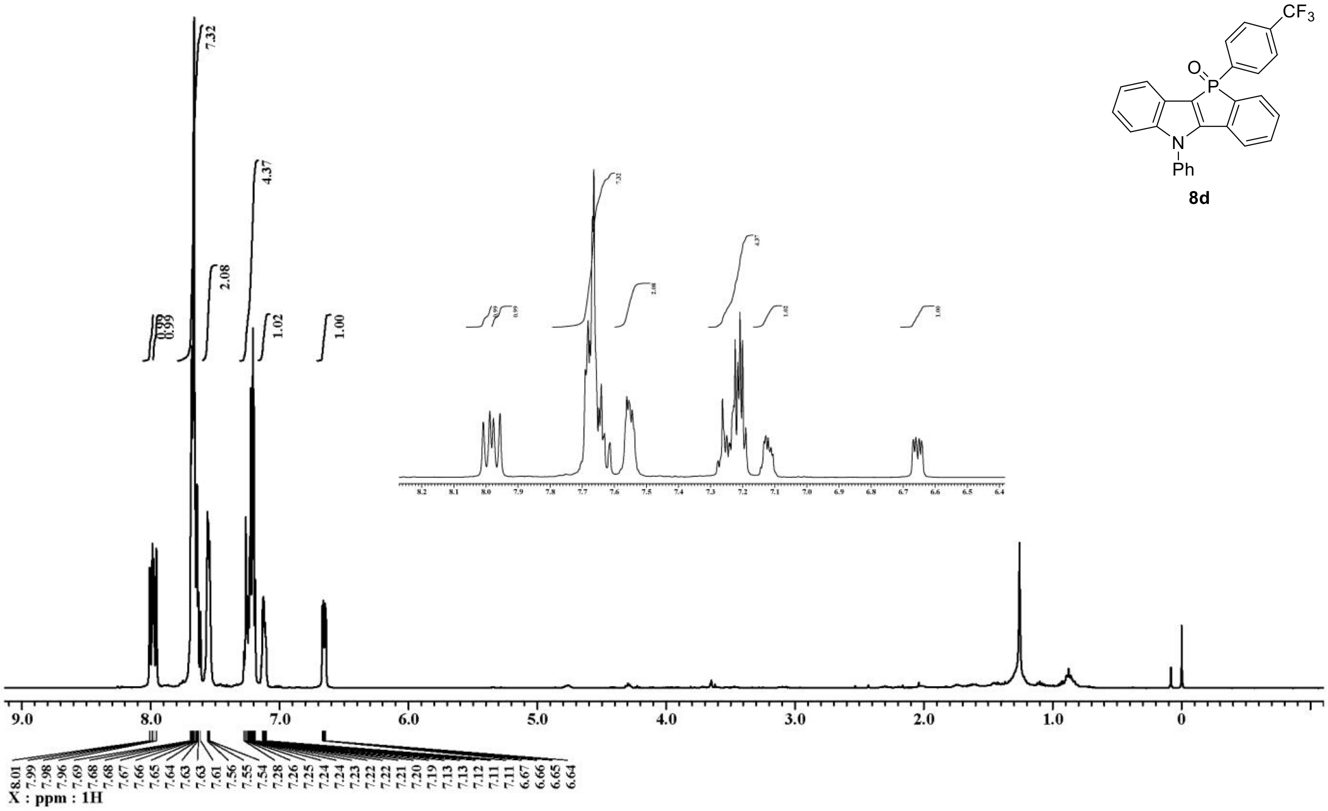

Figure S14. ^{1}H NMR spectrum of **8d** (400 MHz, $CDCl_3$).

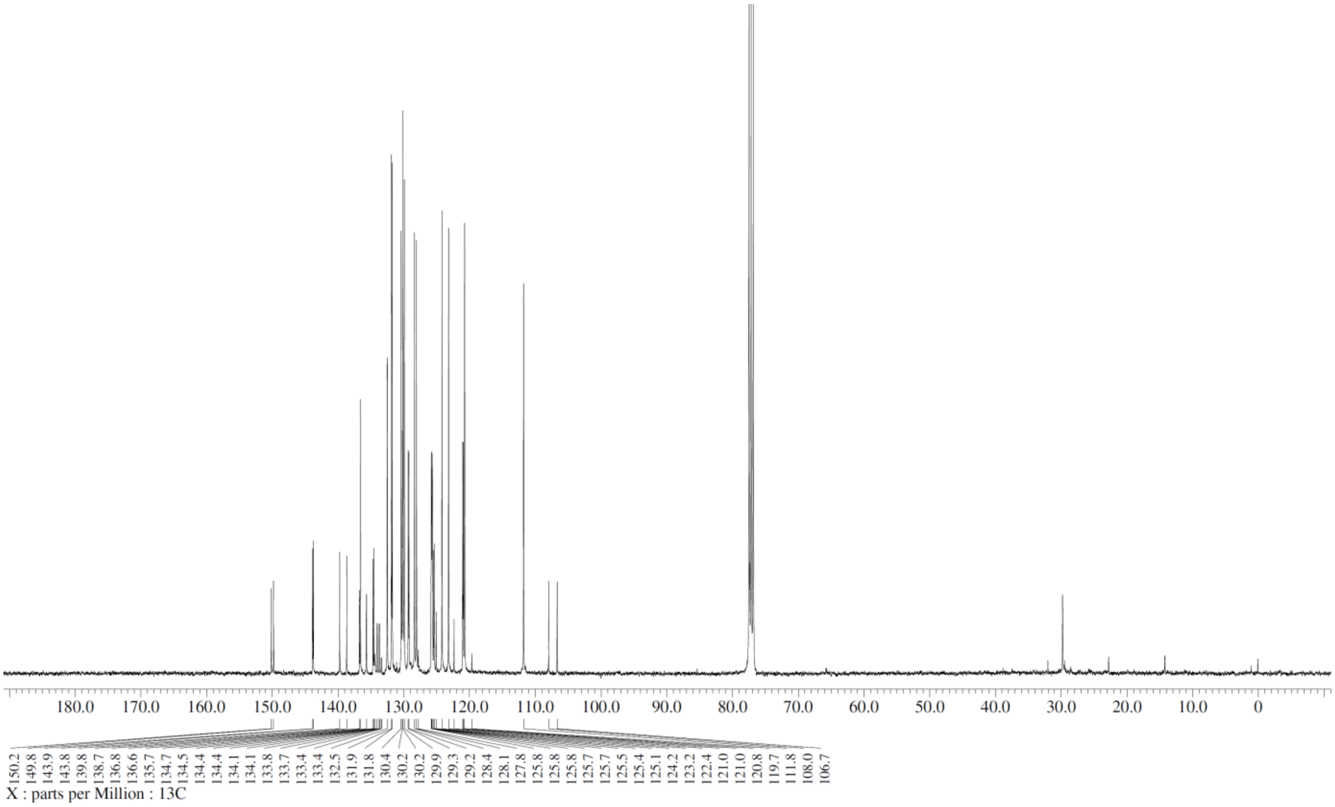

Figure S15. ^{13}C NMR spectrum of **8d** (101 MHz, $CDCl_3$).

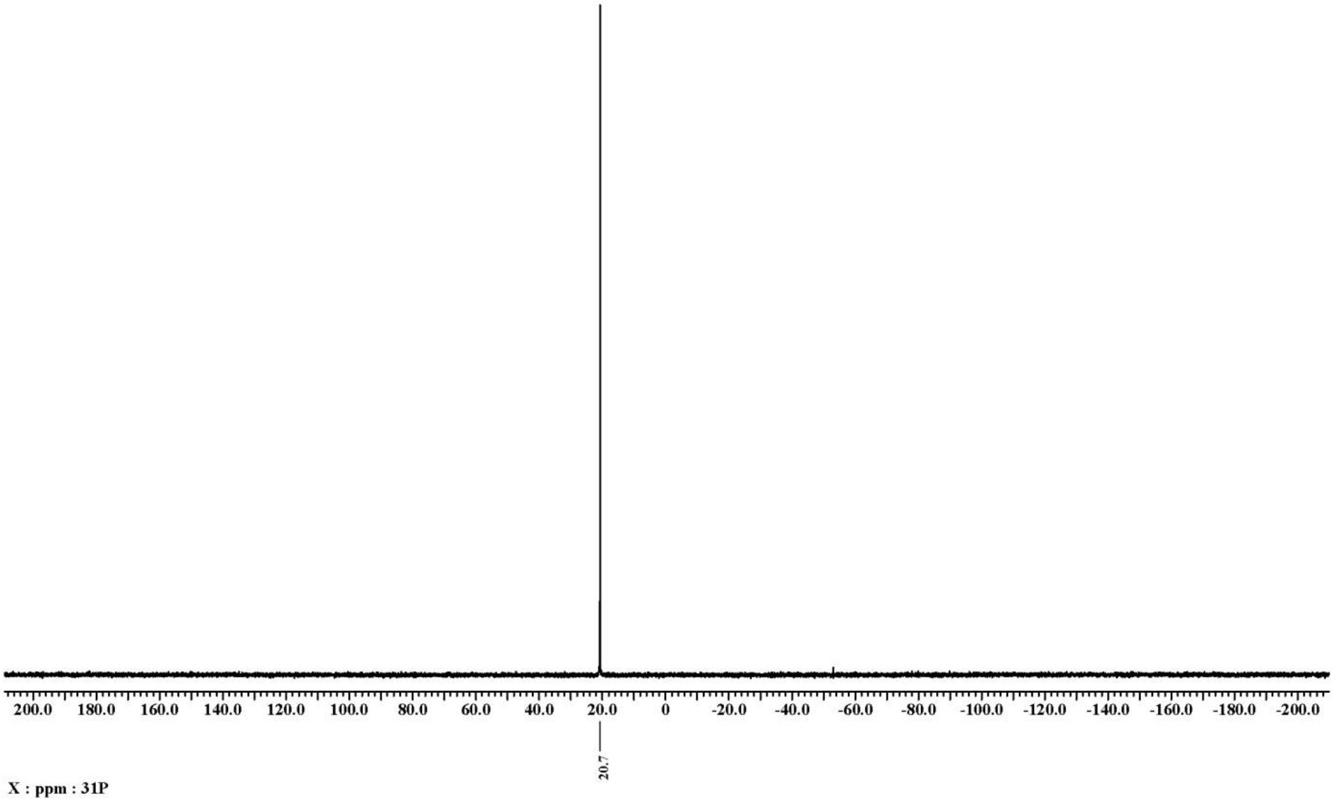

Figure S16. ^{31}P NMR spectrum of **8d** (162 MHz, $CDCl_3$).

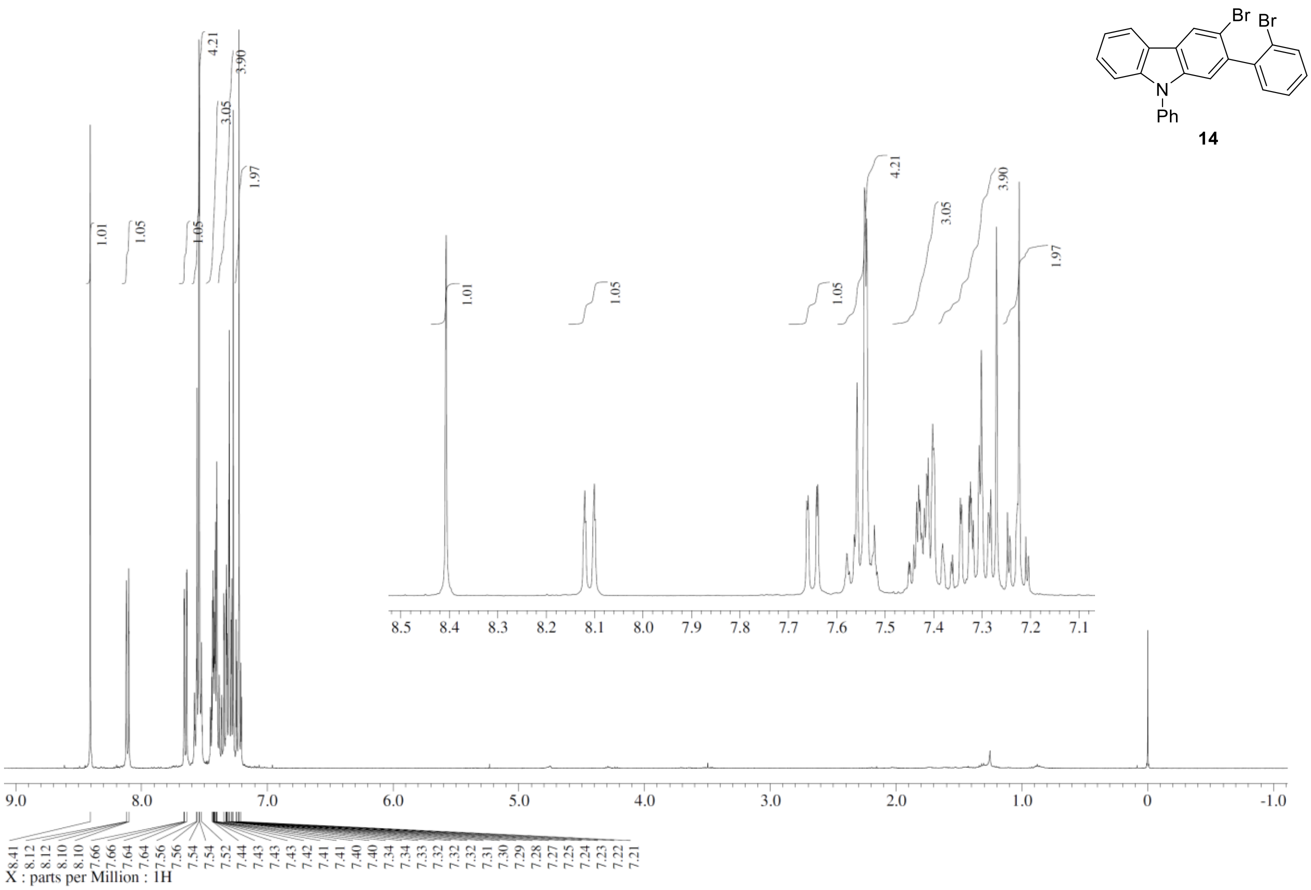

Figure S17. ^{1}H NMR spectrum of **14** (400 MHz, $CDCl_3$).

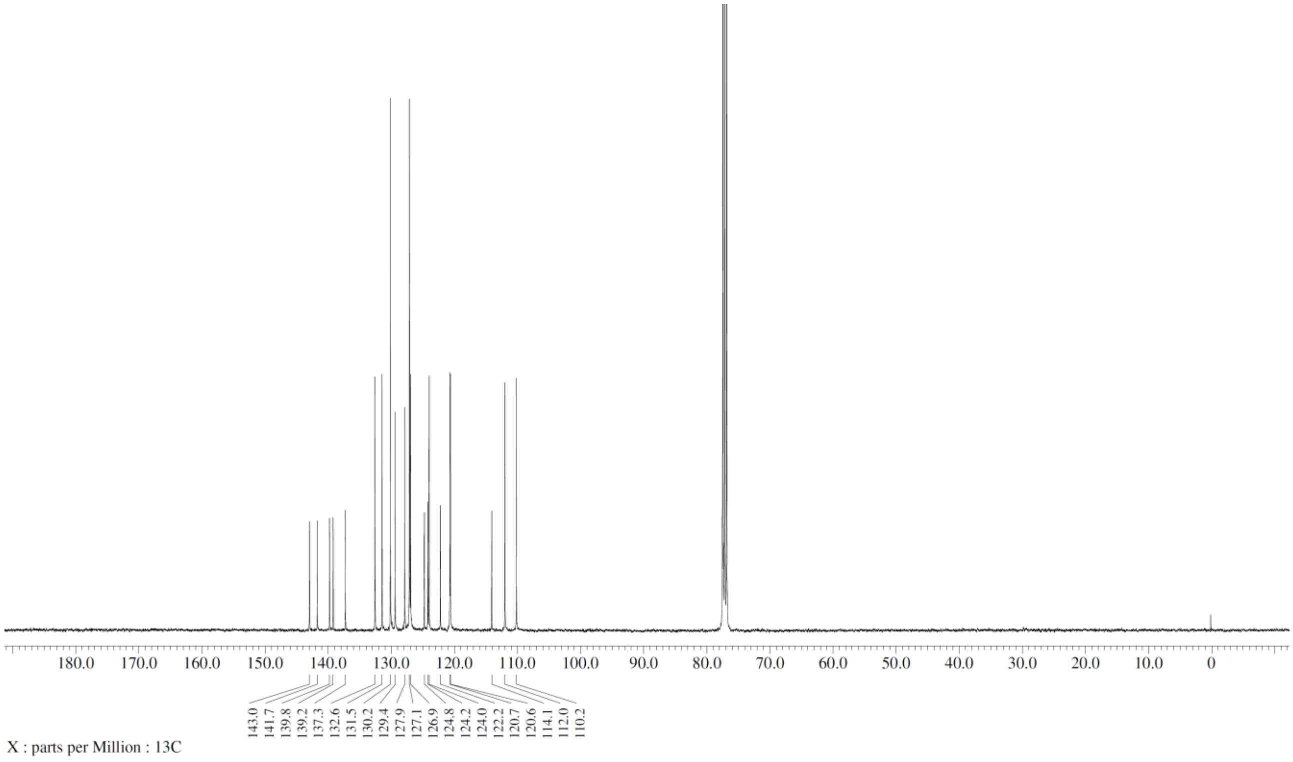

Figure S18. ^{13}C NMR spectrum of **14** (101 MHz, $CDCl_3$).

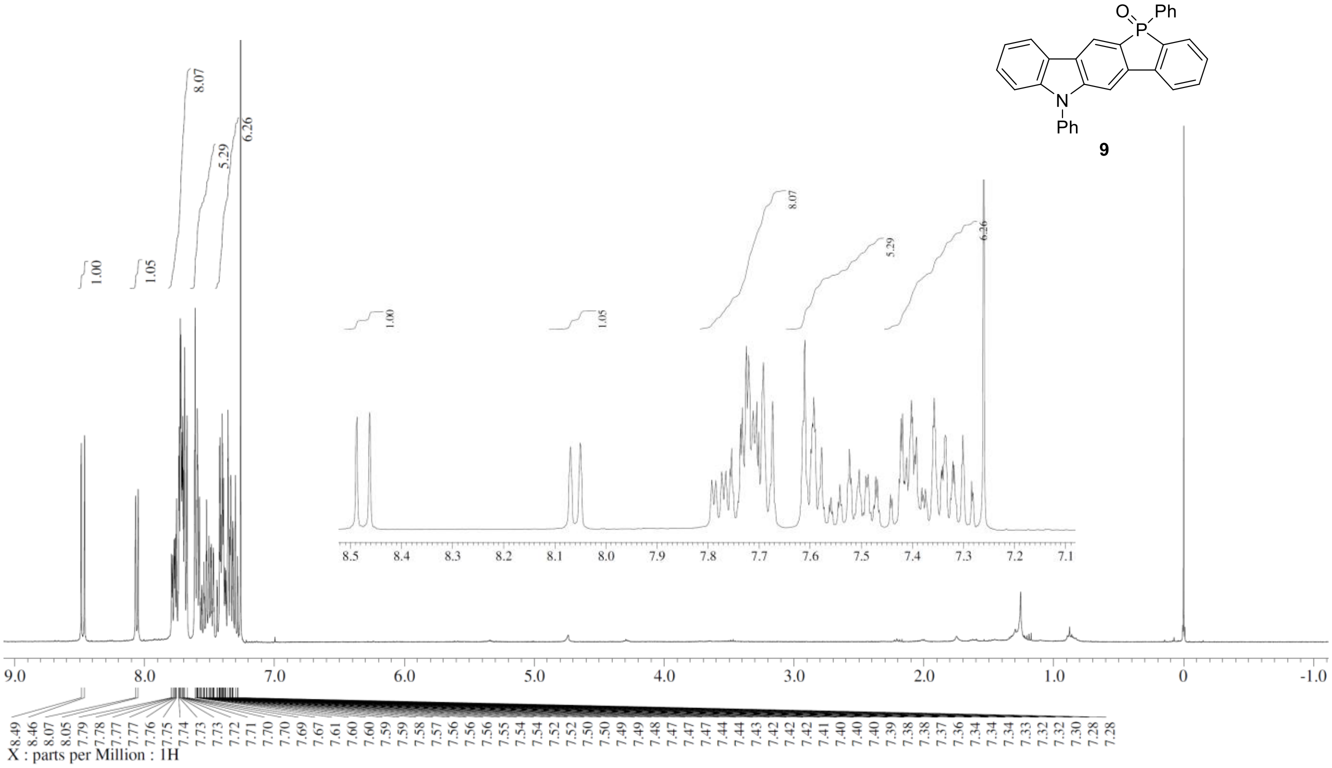

Figure S19. ^{1}H NMR spectrum of **9** (400 MHz, $CDCl_3$).

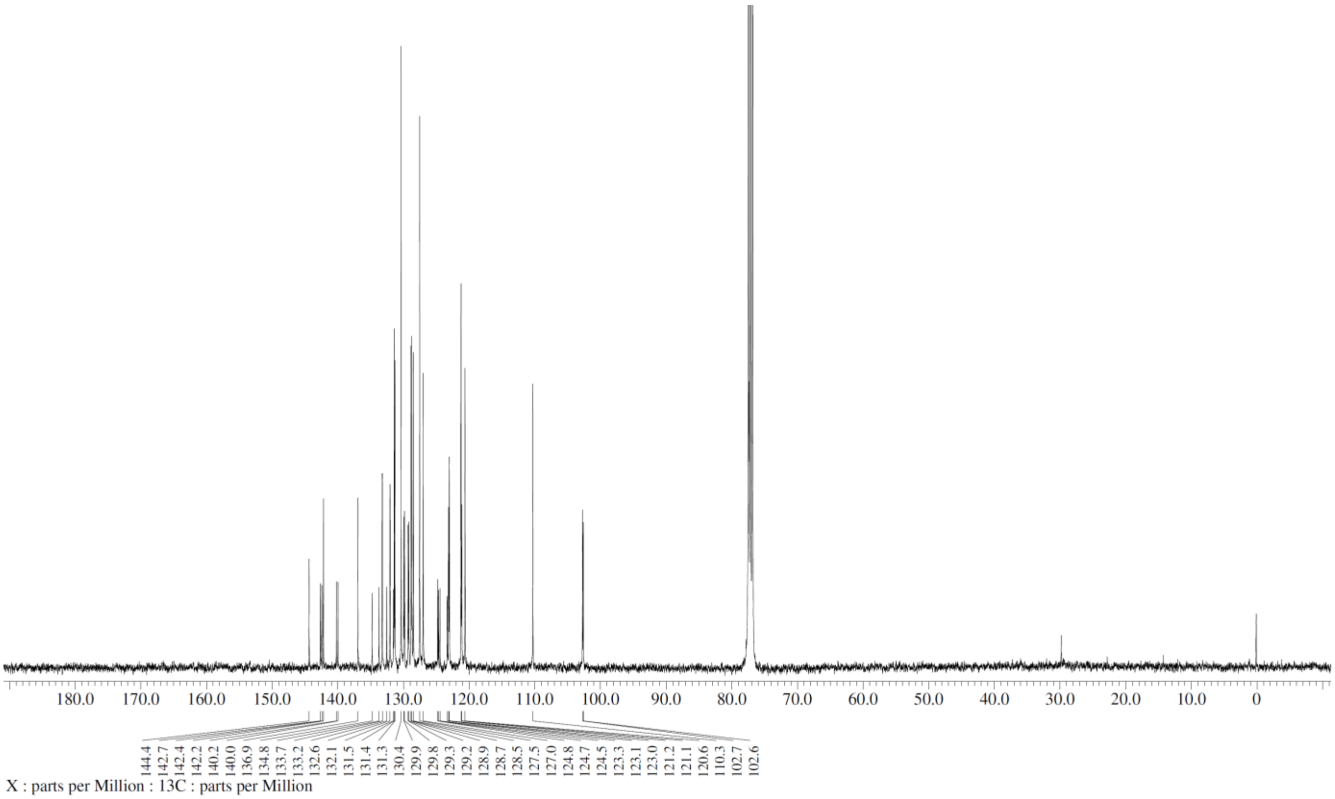

Figure S20. ^{13}C NMR spectrum of **9** (101 MHz, $CDCl_3$).

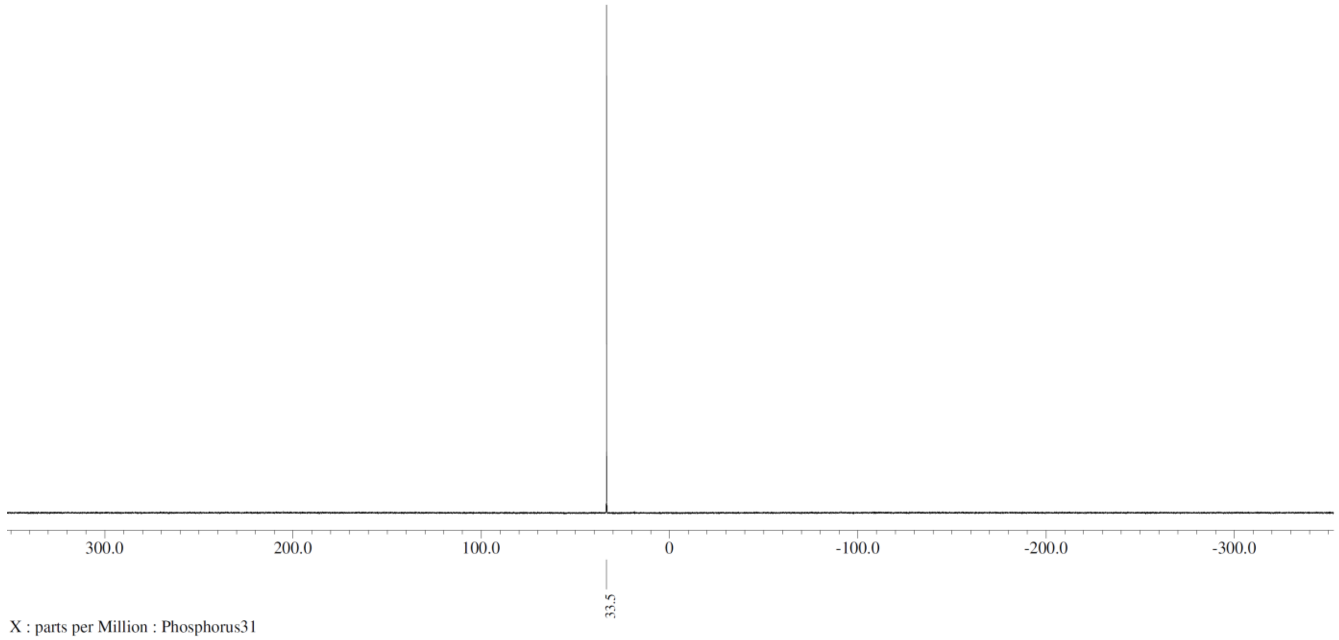

Figure S21. ^{31}P NMR spectrum of **9** (202 MHz, $CDCl_3$).

Table S1. Crystallographic Data and Structure Refinement Details for **8a**

Formula	$C_{26}H_{18}NOP$	
Formula weight	391.38	
Temperature	193(2) K	
Wavelength	1.54187 Å	
Crystal system	monoclinic	
Space group	$P2_1/c$	
Unit cell dimensions	a = 9.0262(2) Å	α = 90°
	b = 10.9540(2) Å	β = 100.0930(10)°
	c = 20.2813(4) Å	γ = 90°
Volume	1974.24(7) Å^3	
Z	4	
Density (calculated)	1.317 g/cm^3	
Absorption coefficient	1.359 mm^{-1}	
F(000)	816	
Crystal size	0.50 × 0.15 × 0.10 mm^3	
Theta range for data collection	4.429 to 68.229°	
Index ranges	–10<=h<=10, –13<=k<=13, –24<=l<=24	
Reflections collected	35030	
Independent reflections	3597 [R_{int} = 0.0354]	
Completeness to theta	99.6%	
Max. and min. transmission	0.873 and 0.613	
Refinement method	Full-matrix least-squares on F^2	
Data / restraints / parameters	3597 / 0 / 262	
Goodness-of-fit on F^2	1.101	
Final R indices [I>2sigma(I)]	R_1 = 0.0412, wR_2 = 0.1160	
R indices (all data)	R_1 = 0.0455, wR_2 = 0.1191	
Largest diff. peak and hole	0.530 and –0.327 e/Å^3	

Table S2. Crystallographic Data and Structure Refinement Details for Racemic **9**

Formula	$C_{30}H_{20}NOP{\cdot}H_2O$	
Formula weight	459.45	
Temperature	193(2) K	
Wavelength	1.54187 Å	
Crystal system	monoclinic	
Space group	*C*2/*c*	
Unit cell dimensions	*a* = 36.7954(7) Å	α = 90°
	b = 6.48420(12) Å	β = 102.4869(8)°
	c = 19.5726(4) Å	γ = 90°
Volume	4559.33(15) $Å^3$	
Z	8	
Density (calculated)	1.339 g/cm^3	
Absorption coefficient	1.294 mm^{-1}	
F(000)	1920	
Crystal size	0.55 × 0.20 × 0.050 mm^3	
Theta range for data collection	4.628 to 68.219°	
Index ranges	–44<=h<=43, –7<=k<=7, –23<=l<=23	
Reflections collected	38357	
Independent reflections	4170 [R_{int} = 0.0292]	
Completeness to theta	100.0%	
Max. and min. transmission	0.937 and 0.632	
Refinement method	Full-matrix least-squares on F^2	
Data / restraints / parameters	4170 / 3 / 313	
Goodness-of-fit on F^2	1.073	
Final *R* indices [*I*>2sigma(*I*)]	R_1 = 0.0502, wR_2 = 0.1424	
R indices (all data)	R_1 = 0.0570, wR_2 = 0.1482	
Largest diff. peak and hole	0.5726 and –0.357 e/$Å^3$	

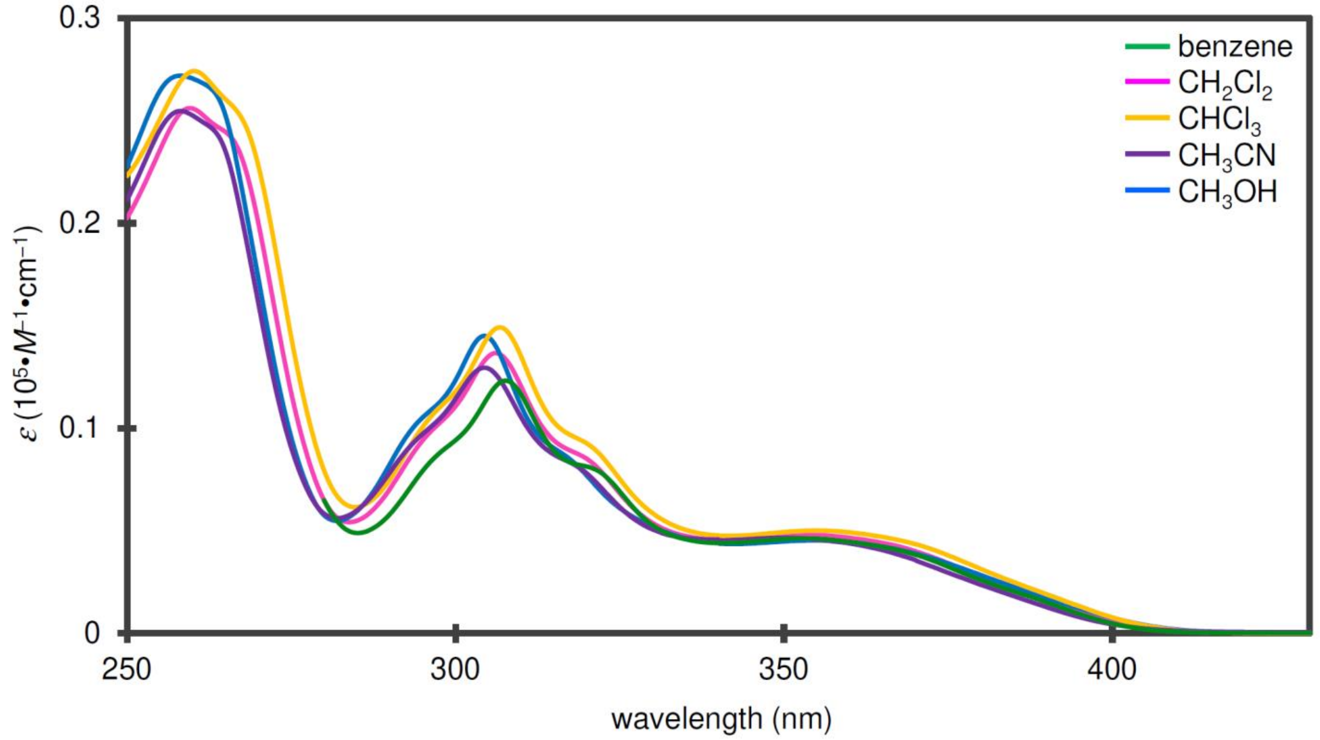

Figure S22. UV/Vis absorption spectra of **8a** in various solvents.

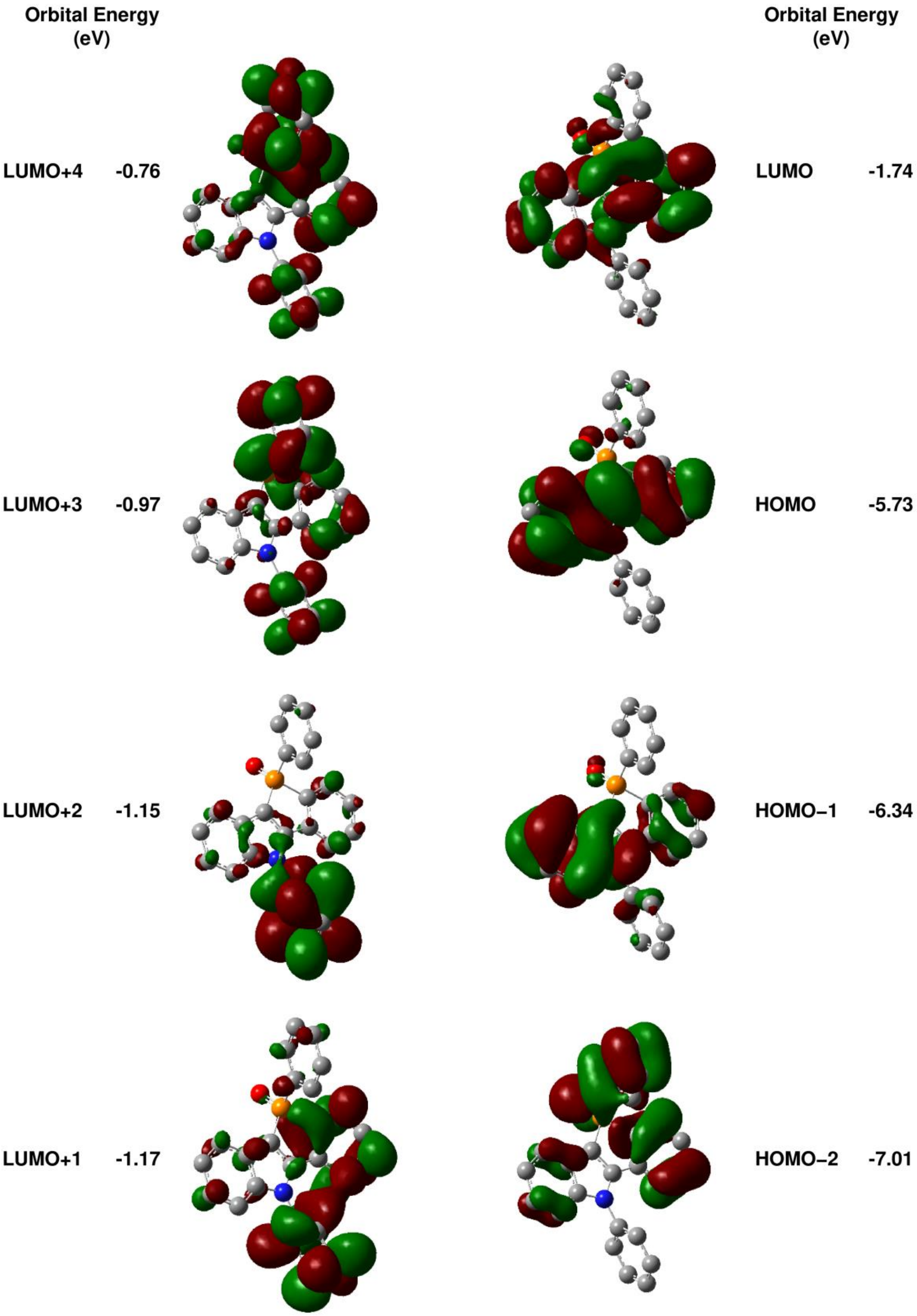

Figure S23. Molecular orbitals of **8a** calculated by DFT method at the B3LYP/6-31G(d) level of theory.

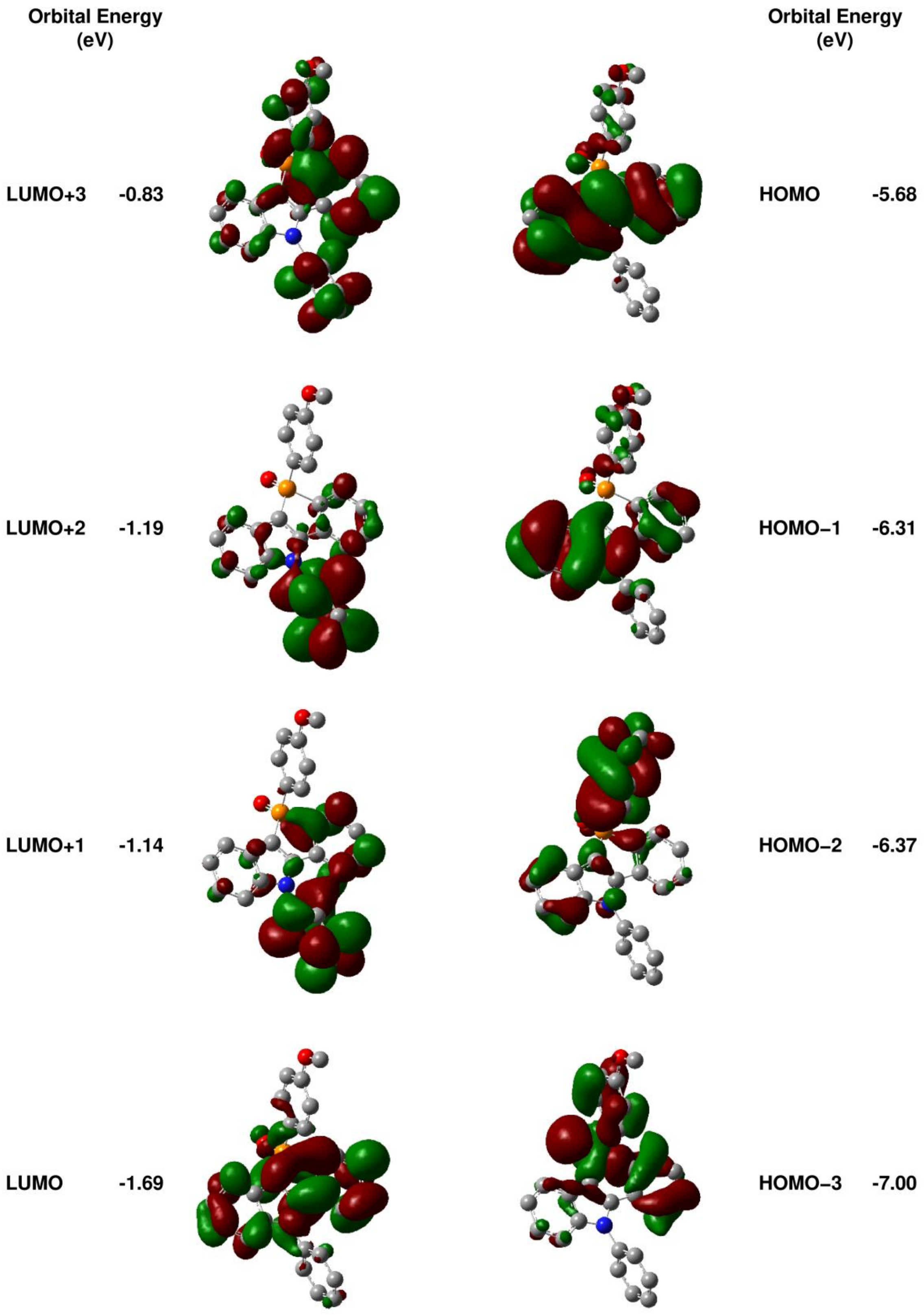

Figure S24. Molecular orbitals of **8b** calculated by DFT method at the B3LYP/6-31G(d) level of theory.

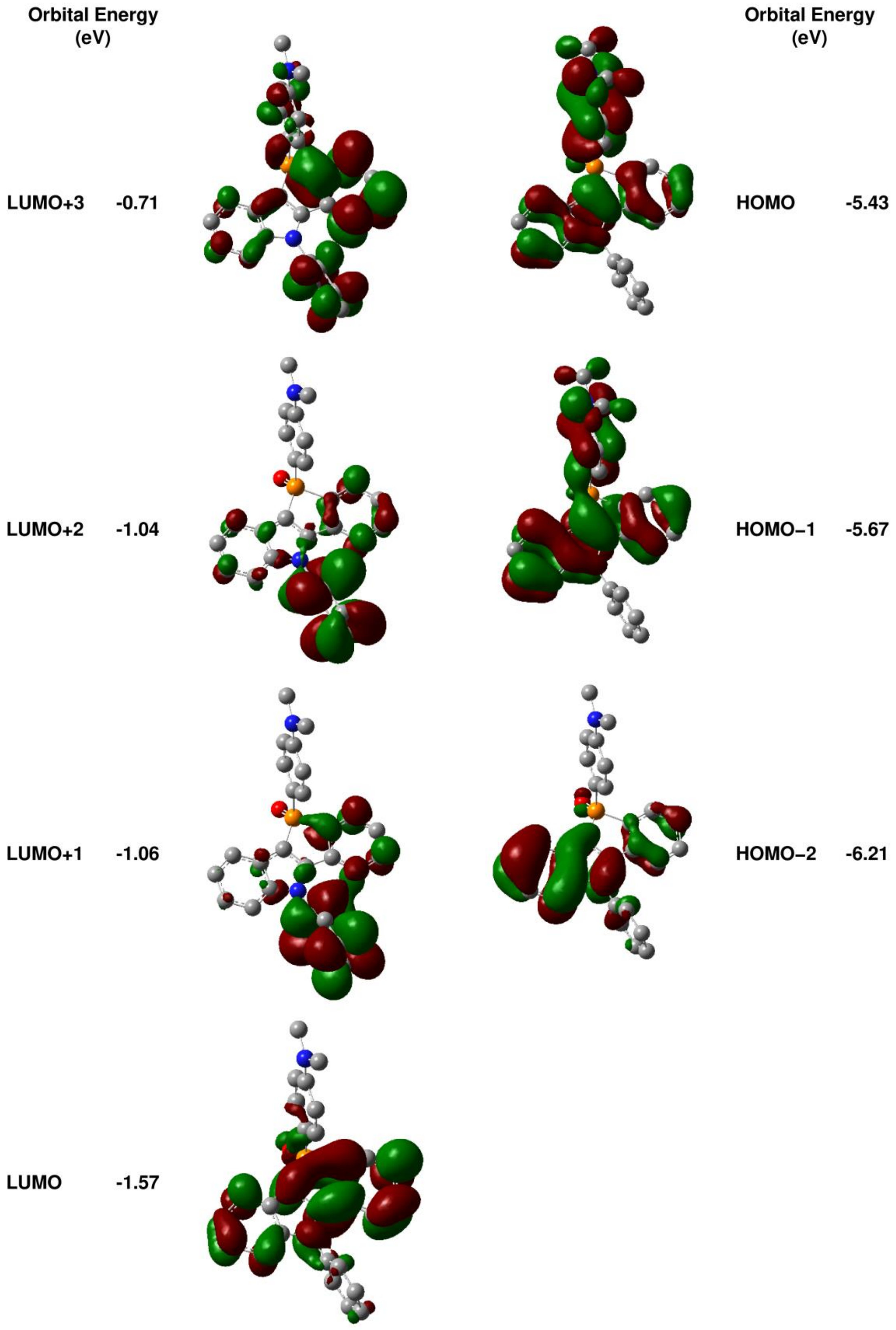

Figure S25. Molecular orbitals of **8c** calculated by DFT method at the B3LYP/6-31G(d) level of theory.

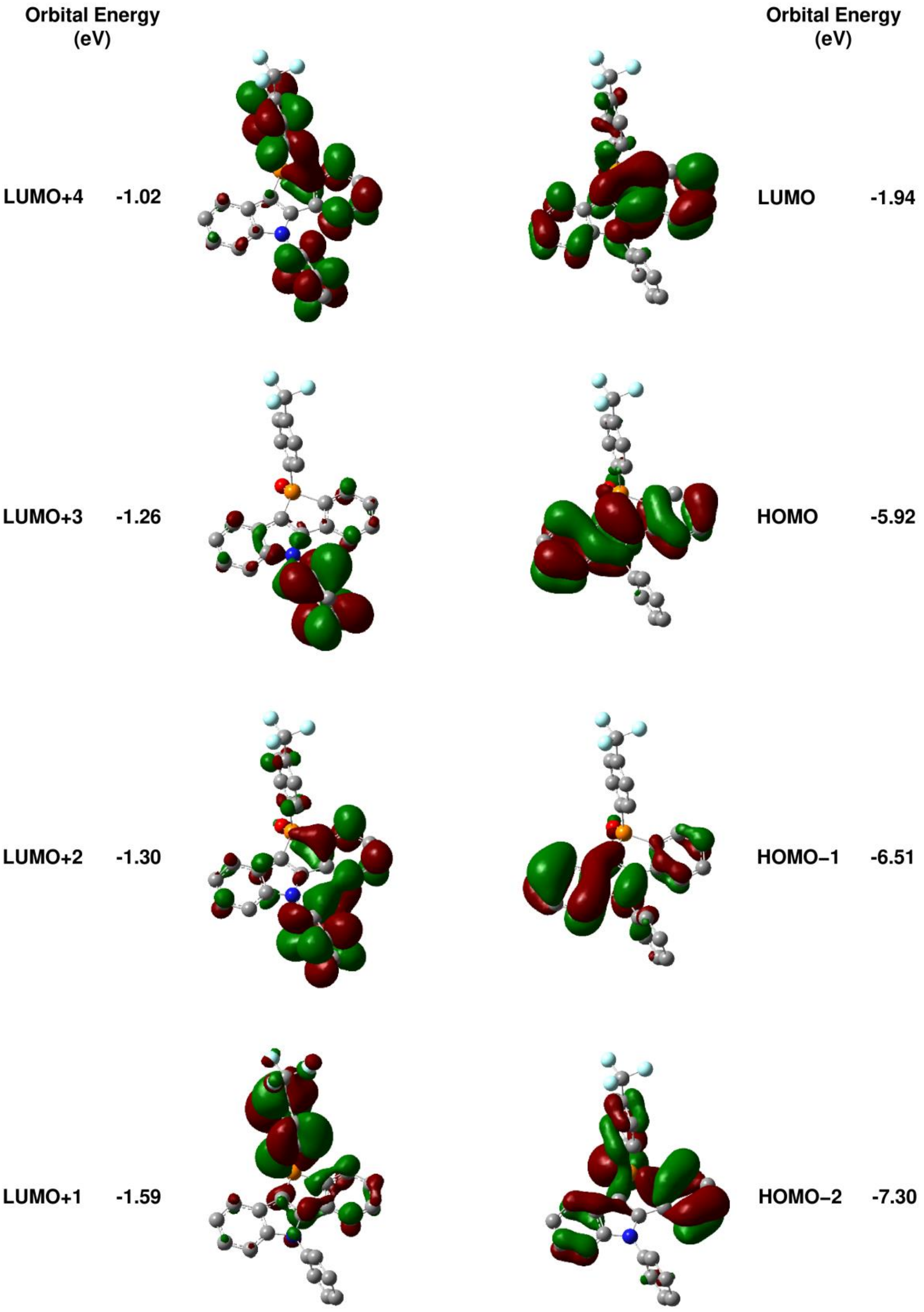

Figure S26. Molecular orbitals of **8d** calculated by DFT method at the B3LYP/6-31G(d) level of theory.

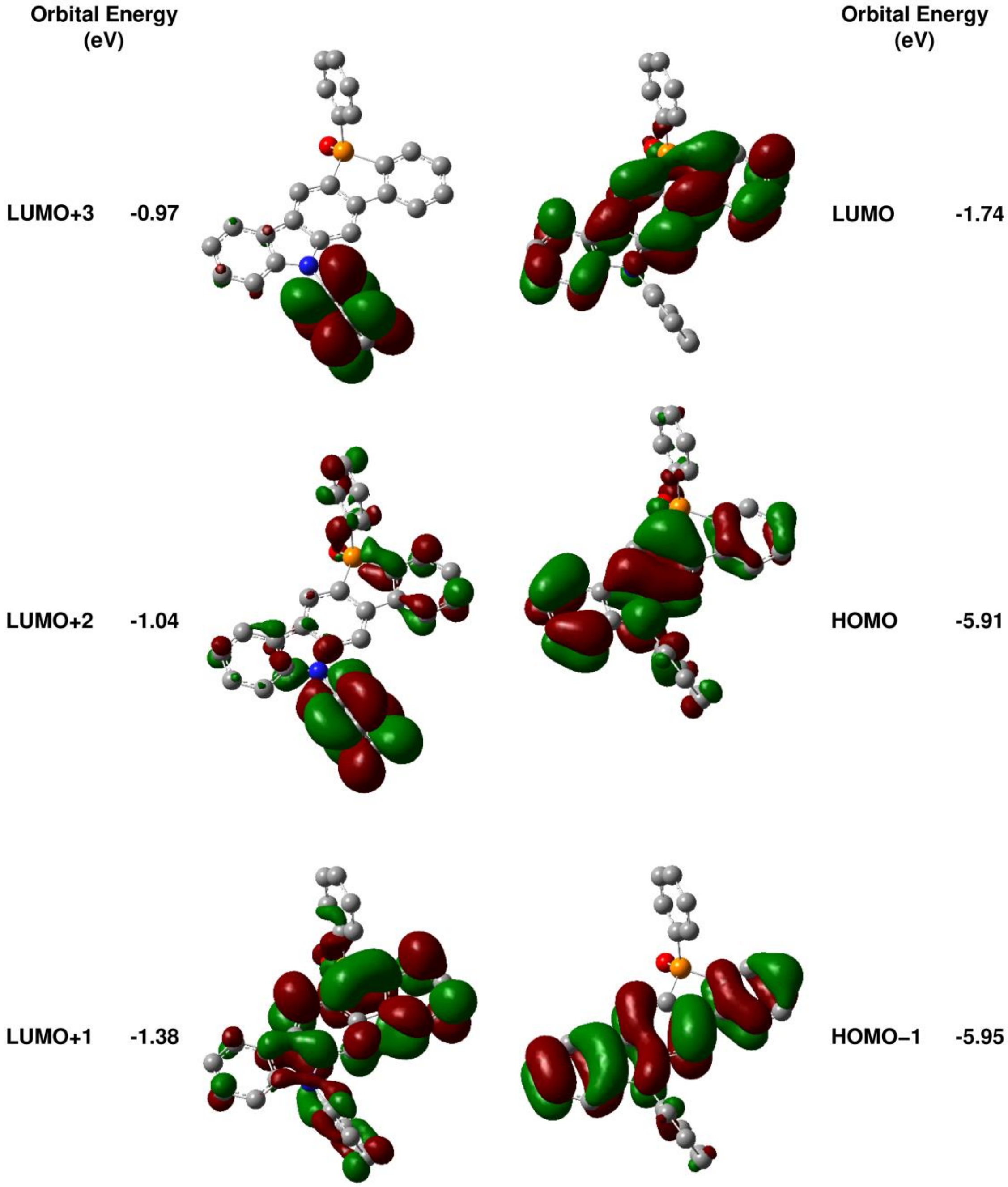

Figure S27. Molecular orbitals of **9** calculated by DFT method at the B3LYP/6-31G(d) level of theory.

Table S3. Coordinates (Å) and Absolute Energy of the Optimized Structure for **8a**[a]

atom	x	y	z	atom	x	y	z
C	-1.4710064	1.8703390	-0.2204162	O	2.5577434	-0.0767658	-2.4563447
C	-0.4362301	4.3964519	-0.6827955	C	3.1377638	0.0605703	0.2406973
C	-0.1114554	2.0106377	-0.6260102	C	5.0874636	0.4808042	2.2078611
C	-2.3167722	2.9701923	-0.0479562	C	4.4811808	0.2259755	-0.1231430
C	-1.7797422	4.2341759	-0.2827804	C	2.7747203	0.1103287	1.5951590
C	0.4008082	3.2998772	-0.8583202	C	3.7466936	0.3180844	2.5748533
H	-3.3520827	2.8451017	0.2556095	C	5.4528910	0.4360014	0.8601663
H	-2.4096534	5.1108769	-0.1579011	H	4.7490065	0.1959189	-1.1756755
H	1.4313147	3.4310972	-1.1762185	H	1.7332092	-0.0080442	1.8846408
H	-0.0524522	5.3975790	-0.8603215	H	3.4593967	0.3573000	3.6226365
C	0.4183693	0.6827965	-0.7045719	H	6.4929663	0.5665853	0.5718928
C	-0.5952978	-0.1950744	-0.3575591	H	5.8431436	0.6452253	2.9720677
N	-1.7470774	0.5061075	-0.0585222	C	-3.0307229	-0.0243811	0.2928425
C	-0.2731818	-1.6285402	-0.3566726	C	-5.5409993	-1.0351033	0.9755138
C	0.7971257	-4.2113760	-0.5366474	C	-3.4281659	-0.0495644	1.6334980
C	-1.0745958	-2.7314560	-0.0498619	C	-3.8874202	-0.4916035	-0.7095420
C	1.0773521	-1.8263068	-0.7413686	C	-5.1411128	-1.0019595	-0.3641449
C	1.6078801	-3.1048928	-0.8372347	C	-4.6854391	-0.5563952	1.9724284
C	-0.5279730	-4.0194510	-0.1425279	H	-2.7515796	0.3222070	2.3978168
H	-2.1077297	-2.6042527	0.2553296	H	-3.5656861	-0.4546882	-1.7464685
H	2.6402701	-3.2493234	-1.1460345	H	-5.8054431	-1.3692773	-1.1417226
H	-1.1509642	-4.8783370	0.0937974	H	-4.9940206	-0.5789085	3.0141640
H	1.2026671	-5.2167910	-0.6114570	H	-6.5175469	-1.4309151	1.2414758
P	1.9219986	-0.2220510	-1.1001347				
absolute energy *E* (B3LYP): -1473.01230255 au							

[a]Calculated by DFT method [B3LYP/6-31G(d)]

Table S4. Coordinates (Å) and Absolute Energy of the Optimized Structure for **8b**[a]

atom	x	y	z	atom	x	y	z
C	1.8747031	-1.8909209	-0.2937698	C	-4.9615238	-0.2689778	1.0206093
C	0.8101112	-4.3104747	-1.1181655	C	-3.9228199	0.1511844	-1.1250466
C	0.6018308	-1.9177341	-0.9355389	C	-2.5585818	-0.0514803	0.8565881
C	2.6209592	-3.0509393	-0.0653838	C	-3.6997146	-0.2649852	1.6339728
C	2.0698550	-4.2602251	-0.4842657	C	-5.0663882	-0.0615075	-0.3637764
C	0.0732963	-3.1538488	-1.3494375	H	-3.9983932	0.3025363	-2.1982167
H	3.5929487	-3.0120817	0.4177436	H	-1.5854723	-0.0583967	1.3420826
H	2.6235950	-5.1811386	-0.3220430	H	-3.5935644	-0.4302007	2.7003063
H	-0.8909286	-3.1980210	-1.8479802	H	-6.0517988	-0.0752582	-0.8201354
H	0.4130685	-5.2712923	-1.4348476	C	3.4184934	-0.1357735	0.6114127
C	0.1616383	-0.5569746	-1.0026015	C	5.8284471	0.6675583	1.7683014
C	1.1416413	0.2302232	-0.4215968	C	3.6014033	-0.2589662	1.9925218
N	2.1867150	-0.5604452	0.0165537	C	4.4400097	0.3765236	-0.1956335
C	0.8952517	1.6776744	-0.3637954	C	5.6426424	0.7833181	0.3870680
C	0.0012706	4.3279066	-0.5207556	C	4.8088910	0.1437610	2.5689573
C	1.6814589	2.7046243	0.1656087	H	2.7988028	-0.6630179	2.6030341
C	-0.3539350	1.9844544	-0.9606602	H	4.2844291	0.4548238	-1.2678956
C	-0.7953717	3.2972326	-1.0453284	H	6.4347029	1.1856811	-0.2387528
C	1.2236269	4.0274402	0.0822312	H	4.9506359	0.0507342	3.6423850
H	2.6358776	2.4926737	0.6355514	H	6.7658858	0.9825944	2.2188728
H	-1.7479316	3.5264985	-1.5167140	O	-6.1383309	-0.4686309	1.6812742
H	1.8352932	4.8274061	0.4916567	C	-6.1075599	-0.6868548	3.0865008
H	-0.3351060	5.3592888	-0.5849882	H	-5.5387481	-1.5916518	3.3370828
P	-1.2012115	0.4641618	-1.5865945	H	-7.1483011	-0.8171938	3.3876303
O	-1.5764659	0.4580913	-3.0444028	H	-5.6796453	0.1752665	3.6144610
C	-2.6522699	0.1620541	-0.5232103				

absolute energy *E* (B3LYP): -1587.38899062 au

[a]Calculated by DFT method [B3LYP/6-31G(d)]

Table S5. Coordinates (Å) and Absolute Energy of the Optimized Structure for **8c**[a]

atom	x	y	z	atom	x	y	z
C	2.1375653	-1.9002404	-0.2751126	C	-2.3248841	-0.0085790	0.7407796
C	1.0739736	-4.3102334	-1.1286029	C	-3.4882095	-0.2117704	1.4740687
C	0.8839581	-1.9150952	-0.9543554	C	-4.7858408	-0.0280429	-0.5619362
C	2.8652763	-3.0673434	-0.0239576	H	-3.6577428	0.3226636	-2.3576356
C	2.3149573	-4.2717375	-0.4579488	H	-1.3721260	-0.0063292	1.2657601
C	0.3558117	-3.1465724	-1.3823704	H	-3.4068486	-0.3576428	2.5447730
H	3.8228277	-3.0375539	0.4878848	H	-5.7273376	-0.0282070	-1.0985207
H	2.8545859	-5.1978910	-0.2785709	C	3.6688093	-0.1595955	0.6768556
H	-0.5940627	-3.1814842	-1.9084227	C	6.0479981	0.6216843	1.9108478
H	0.6767624	-5.2675297	-1.4557600	C	3.8069390	-0.2855445	2.0629837
C	0.4588664	-0.5504653	-1.0361573	C	4.7200574	0.3445969	-0.0964947
C	1.4283273	0.2275578	-0.4269599	C	5.9070819	0.7403909	0.5245631
N	2.4529956	-0.5726814	0.0431267	C	4.9989604	0.1060008	2.6779976
C	1.1933757	1.6770936	-0.3760842	H	2.9816642	-0.6828758	2.6471033
C	0.3280322	4.3349543	-0.5569053	H	4.5990991	0.4254866	-1.1730176
C	1.9732899	2.6968629	0.1763079	H	6.7220420	1.1365809	-0.0753042
C	-0.0353473	1.9944600	-1.0086266	H	5.1056291	0.0108279	3.7553171
C	-0.4622015	3.3113047	-1.1044429	H	6.9733460	0.9281823	2.3913717
C	1.5300575	4.0237732	0.0806360	N	-5.9168653	-0.4679754	1.5591398
H	2.9117557	2.4762385	0.6736647	C	-7.2090348	-0.3138528	0.9088419
H	-1.3994462	3.5487577	-1.6018495	H	-7.3058404	-0.9983631	0.0574844
H	2.1367949	4.8181655	0.5079916	H	-7.3789885	0.7125155	0.5453211
H	0.0025306	5.3693251	-0.6299014	H	-7.9985666	-0.5602209	1.6214230
P	-0.8807832	0.4816362	-1.6595042	C	-5.8703444	-0.4899065	3.0127596
O	-1.2009128	0.4819231	-3.1312217	H	-5.2031516	-1.2816518	3.3749706
C	-2.3617239	0.1893956	-0.6477108	H	-6.8698290	-0.6999873	3.3983673
C	-4.7563626	-0.2368518	0.8387419	H	-5.5301701	0.4678714	3.4388865
C	-3.6121868	0.1757210	-1.2818621				

absolute energy *E* (B3LYP): -1606.83727225 au

[a]Calculated by DFT method [B3LYP/6-31G(d)]

Table S6. Coordinates (Å) and Absolute Energy of the Optimized Structure for **8d**[a]

atom	x	y	z	atom	x	y	z
C	2.3215005	-1.9083689	-0.2713682	C	-2.1234408	0.2417396	-0.7830502
C	1.2803401	-4.3082722	-1.1775432	C	-4.5085044	-0.1485920	0.6237521
C	1.1013716	-1.9128322	-1.0084087	C	-3.3411475	0.1641884	-1.4684505
C	3.0267754	-3.0814388	0.0134990	C	-2.1068915	0.1186935	0.6147355
C	2.4878655	-4.2803902	-0.4478238	C	-3.2930367	-0.0736483	1.3177530
C	0.5842357	-3.1390552	-1.4638323	C	-4.5346134	-0.0303217	-0.7673447
H	3.9584259	-3.0596365	0.5712444	H	-3.3436063	0.2504044	-2.5512164
H	3.0100060	-5.2112125	-0.2435148	H	-1.1659433	0.1671358	1.1565522
H	-0.3390248	-3.1668935	-2.0357389	H	-3.2768917	-0.1722524	2.3991449
H	0.8917433	-5.2619159	-1.5245617	H	-5.4758616	-0.0942315	-1.3032413
C	0.6931697	-0.5436967	-1.1057916	C	3.8182893	-0.1819456	0.7631645
C	1.6408814	0.2259147	-0.4495967	C	6.1332334	0.5778536	2.1229109
N	2.6333004	-0.5836726	0.0640101	C	3.8636163	-0.2681300	2.1582363
C	1.4196898	1.6780157	-0.4116070	C	4.9288933	0.2710528	0.0433857
C	0.5934708	4.3458745	-0.6412207	C	6.0845179	0.6558556	0.7274802
C	2.1860254	2.6911476	0.1703385	C	5.0241383	0.1134409	2.8365090
C	0.2240004	2.0092696	-1.0988343	H	2.9933493	-0.6278840	2.6998955
C	-0.1844188	3.3299533	-1.2197191	H	4.8787239	0.3211326	-1.0406291
C	1.7621893	4.0224300	0.0497538	H	6.9465055	1.0120164	0.1698756
H	3.0991724	2.4618596	0.7089499	H	5.0598283	0.0494401	3.9206967
H	-1.0944608	3.5769489	-1.7608488	H	7.0340948	0.8759493	2.6525728
H	2.3587811	4.8115255	0.5001642	C	-5.7831757	-0.3236950	1.4070067
H	0.2844296	5.3835584	-0.7325960	F	-6.1314783	0.8175662	2.0637065
P	-0.6008770	0.5043747	-1.7779456	F	-5.6673821	-1.2879269	2.3573705
O	-0.9149312	0.4888942	-3.2485618	F	-6.8359865	-0.6604469	0.6255068

absolute energy *E* (B3LYP): -1809.92118229 au

[a]Calculated by DFT method [B3LYP/6-31G(d)]

Table S7. Coordinates (Å) and Absolute Energy of the Optimized Structure for **9**[a]

atom	x	y	z	atom	x	y	z
C	-1.5575491	2.0675064	-0.3603474	C	1.5868930	-0.0622735	-0.2226325
C	-3.6709127	3.9118239	-0.4487146	C	-0.3390704	-1.4349313	-0.8175529
C	-1.3196598	3.4155376	-0.0692667	H	1.2477668	2.0464694	0.1468913
C	-2.8666322	1.6525218	-0.6849710	H	-0.7826115	-2.3910720	-1.0847827
C	-3.9183614	2.5642942	-0.7350044	N	2.9551235	-0.2100418	-0.0067531
C	-2.3773016	4.3289739	-0.1146785	C	2.1260961	-2.2791123	-0.5515900
H	-0.3213178	3.7601486	0.1878514	C	4.6118586	-3.5394553	-0.3865371
H	-4.9199473	2.2339433	-0.9991130	C	2.2179758	-3.6534547	-0.8112061
H	-2.1895319	5.3761951	0.1088647	C	3.2954384	-1.5542979	-0.2041602
H	-4.4820334	4.6340047	-0.4875460	C	4.5476606	-2.1707562	-0.1241673
P	-2.9120479	-0.1427404	-1.0345607	C	3.4609990	-4.2768447	-0.7240429
O	-3.4564970	-0.5944381	-2.3637013	H	1.3328545	-4.2240122	-1.0808470
C	-3.7922983	-0.9504048	0.3552041	H	5.4403343	-1.6067457	0.1280782
C	-5.2108130	-2.2365335	2.3997890	H	3.5459745	-5.3416367	-0.9226724
C	-4.8376835	-1.8294865	0.0415656	H	5.5728640	-4.0441677	-0.3315351
C	-3.4595670	-0.7186712	1.6988744	C	3.8607521	0.8341769	0.343468
C	-4.1671018	-1.3593621	2.7169062	C	5.6388960	2.8812900	1.0304481
C	-5.5445559	-2.4708674	1.0636439	C	4.5267444	0.7981422	1.5748000
H	-5.0845142	-2.0019473	-1.0024047	C	4.0850948	1.8932342	-0.5450502
H	-2.6486239	-0.0395977	1.9519770	C	4.9672715	2.9181570	-0.1952212
H	-3.9051333	-1.1762026	3.7559894	C	5.4198486	1.8179799	1.9114088
H	-6.3542428	-3.1522425	0.8145113	H	4.3371438	-0.0227194	2.2605639
H	-5.7606851	-2.7352011	3.1941929	H	3.5747757	1.9034038	-1.5040071
C	-0.5605093	0.9700077	-0.3848031	H	5.1379522	3.7390382	-0.8867648
C	1.0344344	-1.3290853	-0.5628688	H	5.9365979	1.7856601	2.8669925
C	-1.1217473	-0.2903357	-0.7271822	H	6.3299795	3.6763682	1.2971964
C	0.8036712	1.0934293	-0.1224684				

absolute energy *E* (B3LYP): -1626.67560236 au

[a]Calculated by DFT method [B3LYP/6-31G(d)]

Table S8. The Selected Absorption Peaks of **8a**–**8d** and **9** Calculated by TD−DFT Method at the B3LYP /6-31G(d) Level of Theory.

	excited state	transition energy (eV)	wavelength (nm)	main transition configuration (CI expansion coefficient)	oscillator strength f	Rotatory Strength (10^{-40} erg·esu·cm/gauss)		transition electric dipole moments (a.u.)			transition magnetic dipole moments (a.u.)		
						$R_{velocity}$	R_{length}	x	y	z	x	y	z
**8a**	1	3.4433	360	HOMO → LUMO (0.68476)<br>HOMO → LUMO+1 (0.10607)	0.1105	6.3520	8.1325	0.0150	-1.1415	0.0817	-0.2177	-0.0665	-1.3110
	2	3.9346	315	HOMO−1 → LUMO (-0.23287)<br>HOMO → LUMO+1 (0.57599)<br>HOMO → LUMO+2 (0.31321)	0.0177	9.7538	9.0276	0.3489	0.2411	-0.0637	-0.1383	-0.0073	-0.1839
	3	3.9718	312	HOMO−1 → LUMO (0.26235)<br>HOMO → LUMO+1 (-0.20057)<br>HOMO → LUMO+2 (0.60971)	0.0153	-6.4182	-6.8829	-0.2141	-0.3324	0.0336	-0.0129	-0.0837	-0.0410
	4	4.1106	302	HOMO−1 →LUMO (0.56695)<br>HOMO → LUMO+1 (0.31589)<br>HOMO → LUMO+2 (-0.14102)<br>HOMO → LUMO+3 (0.13998)<br>HOMO → LUMO+4 (0.13063)	0.1720	26.4575	27.7944	-0.7566	1.0623	0.0819	-0.1289	-0.2002	-0.0331
	5	4.1788	297	HOMO−2 → LUMO (-0.11691)<br>HOMO−1 → LUMO (-0.10846)<br>HOMO → LUMO+3 (0.67404)	0.0490	-37.1158	-38.4116	-0.0807	-0.6803	-0.0971	0.1282	-0.2576	0.0204
	6	4.3457	285	HOMO−2 → LUMO (-0.21993)<br>HOMO−1 → LUMO (-0.12160)<br>HOMO−1 → LUMO+1 (0.11174)<br>HOMO → LUMO+4 (0.62876)	0.0655	31.7814	32.4171	0.4737	-0.6172	0.0972	0.1012	0.3312	0.1950
**8b**	1	3.4367	361	HOMO → LUMO (0.68627)	0.1078	17.2955	20.0344	-0.0079	-1.1174	-0.1785	-0.3940	-0.1430	1.3888
	2	3.9107	317	HOMO−1 → LUMO (0.20847)<br>HOMO → LUMO+1 (0.63856)<br>HOMO → LUMO+2 (-0.18047)	0.0136	9.9246	9.0017	-0.2974	-0.1915	-0.1302	0.1955	0.0103	-0.1684
	3	3.9541	314	HOMO−2 → LUMO (0.10186)<br>HOMO−1 → LUMO (0.24718)<br>HOMO → LUMO+2 (0.63765)	0.0095	-0.1215	-0.4461	-0.0913	-0.2876	-0.0808	-0.0141	-0.0269	0.0881
	4	4.1067	302	HOMO−2 → LUMO (0.64012)<br>HOMO−1 → LUMO (-0.25979)	0.0679	12.4081	13.4517	0.7501	-0.3300	0.0596	0.0758	0.3660	0.1157
	5	4.1069	302	HOMO−2 → LUMO (0.20435)<br>HOMO−1 → LUMO (0.50237)<br>HOMO → LUMO+1 (-0.25914)<br>HOMO → LUMO+2 (-0.21463)<br>HOMO → LUMO+3 (0.27463)	0.1059	13.5996	14.5772	-0.4206	0.9290	-0.1126	-0.1470	-0.1457	-0.1040
	6	4.2754	290	HOMO−3 → LUMO (-0.23355)<br>HOMO−2 → LUMO (-0.14747)<br>HOMO−1 → LUMO (-0.20379)<br>HOMO−1 → LUMO+1 (-0.11718)<br>HOMO → LUMO+3 (0.57425)	0.1359	-43.5298	-44.9988	0.0161	-1.1389	0.0210	0.2963	-0.1641	-0.0360

	excited state	transition energy (eV)	wavelength (nm)	main transition configuration (CI expansion coefficient)	oscillator strength f	Rotatory Strength (10^{-40} erg·esu·cm/gauss)		transition electric dipole moments (a.u.)			transition magnetic dipole moments (a.u.)		
						$R_{velocity}$	R_{length}	x	y	z	x	y	z
**8c**	1	3.3214	373	HOMO → LUMO (0.69828)	0.0441	57.1514	60.0651	0.2045	-0.6860	-0.1730	-0.3482	-0.0069	1.0891
	2	3.6098	343	HOMO−1 → LUMO (0.67619)	0.0774	-51.4717	-51.7463	-0.2454	-0.9045	-0.0636	-0.2412	-0.2441	0.9500
	3	3.8482	322	HOMO−2 → LUMO (0.10074)<br>HOMO−1 → LUMO+1 (0.32995)<br>HOMO−1 → LUMO+2 (0.11587)<br>HOMO → LUMO+1 (0.58009)<br>HOMO → LUMO+2 (0.14805)	0.0133	10.5858	9.9489	-0.2940	-0.2166	-0.0867	0.1993	-0.0267	-0.1223
	4	3.9025	318	HOMO−2 → LUMO (-0.16581)<br>HOMO−1 → LUMO (-0.11608)<br>HOMO−1 → LUMO+2 (0.28950)<br>HOMO → LUMO+1 (-0.13780)<br>HOMO → LUMO+2 (0.58498)	0.0391	-10.0456	-11.3862	0.2511	0.5851	0.0572	-0.0380	0.1167	-0.1830
	5	4.0939	303	HOMO−2 → LUMO (-0.16581)<br>HOMO−1 → LUMO+3 (-0.11608)<br>HOMO → LUMO+1 (0.28950)<br>HOMO → LUMO+2 (-0.13780)<br>HOMO → LUMO+3 (0.58498)	0.1120	29.0849	31.0818	-0.8008	0.6716	-0.1569	-0.0872	-0.3479	-0.2037
	6	4.1371	300	HOMO−1 → LUMO+1 (0.48710)<br>HOMO−1 → LUMO+2 (-0.30171)<br>HOMO → LUMO+1 (-0.21828)<br>HOMO → LUMO+2 (0.10099)<br>HOMO → LUMO+3 (-0.32828)	0.0158	-7.2408	-7.6777	0.3757	0.1028	-0.0645	0.0018	0.2533	-0.0907
**8d**	1	3.4137	363	HOMO → LUMO (0.68483)	0.0957	22.9485	26.2524	-0.0175	1.0565	0.1667	0.4691	0.1273	-1.4259
	2	3.7737	329	HOMO → LUMO+1 (0.69452)	0.0459	-50.5408	-51.6931	-0.4534	-0.5394	-0.0076	-0.0232	-0.3916	0.3282
	3	3.9631	313	HOMO−1 → LUMO (-0.46478)<br>HOMO → LUMO+2 (0.50693)	0.0273	4.9992	4.5972	-0.4732	0.0248	-0.2386	0.1174	-0.1069	-0.1622
	4	4.0229	308	HOMO−1 → LUMO (-0.20927)<br>HOMO → LUMO+2 (-0.18012)<br>HOMO → LUMO+3 (0.64678)	0.0018	-0.4392	-0.5711	-0.0897	0.0936	-0.0431	-0.1327	-0.0879	0.0291
	5	4.1183	301	HOMO−1 → LUMO (0.45143)<br>HOMO → LUMO+2 (0.42873)<br>HOMO → LUMO+3 (0.26903)	0.1906	40.8817	42.8672	0.5718	-1.2466	0.0903	0.2671	0.2933	0.3436
	6	4.3054	288	HOMO−2 → LUMO (-0.19646)<br>HOMO−1 → LUMO (0.10331)<br>HOMO → LUMO+4 (0.64315)	0.0608	20.1165	20.6421	0.1705	-0.7383	-0.0441	0.2210	0.1635	0.1028

	excited state	transition energy (eV)	wavelength (nm)	main transition configuration (CI expansion coefficient)	oscillator strength f	Rotatory Strength (10^{-40} erg·esu·cm/gauss)		transition electric dipole moments (a.u.)			transition magnetic dipole moments (a.u.)		
						$R_{velocity}$	R_{length}	x	y	z	x	y	z
**9**	1	3.5895	345	HOMO−1 → LUMO (-0.32279)<br>HOMO−1 → LUMO+1 (-0.15604)<br>HOMO → LUMO (0.56562)<br>HOMO → LUMO+1 (-0.18078)	0.0293	-4.8971	-4.7412	0.4362	0.3562	0.1289	-0.0154	0.1647	-0.2470
	2	3.7078	334	HOMO−1 → LUMO (0.48170)<br>HOMO → LUMO (0.37197)<br>HOMO → LUMO+1 (0.33482)	0.2091	3.8291	4.1979	-1.0789	1.0656	0.0503	-0.0055	0.0166	-0.8230
	3	3.9769	312	HOMO−1 → LUMO (-0.11786)<br>HOMO−1 → LUMO+1 (0.65310)<br>HOMO → LUMO (0.13376)	0.0093	-1.5259	-0.2731	0.2537	-0.1745	0.0197	0.2663	0.2539	-1.1227
	4	4.0589	305	HOMO−1 → LUMO (-0.29656)<br>HOMO−1 → LUMO+2 (0.15573)<br>HOMO → LUMO+1 (0.52207)<br>HOMO → LUMO+2 (-0.28977)	0.5015	18.4539	19.4328	2.1417	-0.6667	0.1073	0.0909	0.3417	-0.4588
	5	4.2762	290	HOMO−1 → LUMO (-0.19349)<br>HOMO−1 → LUMO+1 (-0.12472)<br>HOMO−1 → LUMO+2 (-0.29833)<br>HOMO → LUMO+1 (0.24870)<br>HOMO → LUMO+2 (0.52533)	0.2995	18.8588	19.2513	1.0349	-1.3300	-0.1382	0.2099	0.1556	0.6651
	6	4.2824	290	HOMO−1 → LUMO+3 (-0.35402)<br>HOMO →LUMO+3 (0.59533)	0.0061	-4.0853	1.8358	-0.0785	0.1935	-0.1191	0.3125	-0.2699	-0.5791
